# Molecular Aspects of the Interactions between Selected Benzodiazepines and Common Adulterants/Diluents: Forensic Application of Theoretical Chemistry Methods

**DOI:** 10.3390/ijms251810087

**Published:** 2024-09-19

**Authors:** Jelica Džodić, Milica Marković, Dejan Milenković, Dušan Dimić

**Affiliations:** 1 Faculty of Physical Chemistry, University of Belgrade, Studentski trg 12-16, 11000 Belgrade, Serbia; 2 Department of Science, Institute for Information Technologies, University of Kragujevac, Jovana Cvijića bb, 34000 Kragujevac, Serbia

**Keywords:** benzodiazepines, adulterants, DFT, QTAIM, psychoactive substances, IR

## Abstract

Benzodiazepines are frequently encountered in crime scenes, often mixed with adulterants and diluents, complicating their analysis. This study investigates the interactions between two benzodiazepines, lorazepam (LOR) and alprazolam (ALP), with common adulterants/diluents (paracetamol, caffeine, glucose, and lactose) using infrared (IR) spectroscopy and quantum chemical methods. The crystallographic structures of LOR and ALP were optimized using several functionals (B3LYP, B3LYP-D3BJ, B3PW91, CAM-B3LYP, M05-2X, and M06-2X) combined with the 6-311++G(d,p) basis set. M05-2X was the most accurate when comparing experimental and theoretical bond lengths and angles. Vibrational and ^13^C NMR spectra were calculated to validate the functional’s applicability. The differences between LOR’s experimental and theoretical IR spectra were attributed to intramolecular interactions between LOR monomers, examined through density functional theory (DFT) optimization and quantum theory of atoms in molecules (QTAIM) analysis. Molecular dynamics simulations modeled benzodiazepine–adulterant/diluent systems, predicting the most stable structures, which were further analyzed using QTAIM. The strongest interactions and their effects on IR spectra were identified. Comparisons between experimental and theoretical spectra confirmed spectral changes due to interactions. This study demonstrates the potential of quantum chemical methods in analyzing complex mixtures, elucidating spectral changes, and assessing the structural stability of benzodiazepines in forensic samples.

## 1. Introduction

Benzodiazepines (BDZs) are widespread compounds used for the treatment of mental disorders and are known as anxiolytic drugs. They are depressants that produce sedation and hypnosis, relieve anxiety and muscle spasms, and reduce seizures [1] through the enhancement of gamma-aminobutyric acid effects [2]. They slow down the central nervous system and may cause sleepiness and a relaxed mood. Benzodiazepines are associated with amnesia, hostility, irritability, and vivid or disturbing dreams. The most common benzodiazepines are the prescription drugs Valium^®^, Xanax^®^, Halcion^®^, Ativan^®^, and Lorazepam^®^.

Abuse of benzodiazepines is frequently associated with adolescents and young adults who take the drug orally or crush it up and snort it to feel euphoria, relaxation, or anxiety relief. Abuse is exceptionally high among heroin and cocaine users. Additionally, opioid users often co-abuse benzodiazepines to enhance euphoria. Classical and designer benzodiazepines are becoming more and more present in the misused formulations [3,4]. Although beneficial in supervised use, benzodiazepines lead to drowsiness, confusion, impaired coordination, and memory-related problems [5]. Their use in prolonged periods leads to tolerance, dependence, and withdrawal symptoms. Combined use of benzodiazepines with other central nervous system (CNS) depressants leads to intoxication, fatalities [6], and suicide attempts [7,8].

Alprazolam (ALP) is one of the most commonly prescribed benzodiazepines due to its effect in treating anxiety, alcohol withdrawal syndrome, and nausea [9,10,11]. Due to its extensive consumption in everyday life, ALP is affecting the ecosystem through its presence in wastewater and sewage treatment plants [12]. In addition to its pronounced biological activity examined extensively in [13,14,15], Ildiz and coworkers investigated the molecular structure; IR, UV–Vis, mass, and NMR spectra; photochemical behavior; and stabilization interactions of alprazolam [16]. Lorazepam (LOR) is another widely used benzodiazepine as a sedative and anxiolytic drug [11]. It has antiseizure activity by increasing the opening frequency of gamma-aminobutyric acid–activated chloride channels [17].

Adding substances (bulking) is a common practice in various stages of the production and sale of controlled psychoactive substances [18]. The bulking substances include adulterants and diluents [19]. Adulterants are active substances that enhance the psychotropic effect or minimize the unwanted effects. Diluents include compounds that are added to increase the amount of sold drugs. The most common adulterants/diluents include caffeine, paracetamol, procaine, sugars (lactose, dextrose, mannitol, glucose), and other psychoactive compounds (papaverine, fentanyl, codeine, thebaine) [20,21]. Adulterants are essential in the characterization and profiling of compounds, influencing the solubility and other physical and chemical properties of samples [22]. These compounds are usually determined by gas chromatography, infrared, and Raman spectroscopy, with the extensive use of chemometrics [22,23,24]. Density functional theory (DFT) methods also show specific potential in assigning spectra of novel psychoactive substances in complex mixtures [25,26]. The interactions between adulterants and psychoactive substances can also be necessary for the development of side effects, metabolism, and excretion [27]. The interactions between psychoactive substances and other compounds, like solvent molecules, have been examined by various theoretical chemistry methods.

The interactions between benzodiazepines and caffeine were examined in several studies, and changes in the pharmacokinetics and pharmacodynamics of the processes were observed. Lau and coworkers showed that the ALP pharmacokinetics was not altered by caffeine. Still, caffeine absorption was slower in the presence of ALP, and the ALP/caffeine potency ratio decreased over time [28]. In another study, ALP and caffeine antagonized the behavioral effects of each other when administered to mice, and it was discussed that other sites except benzodiazepine receptors might be included in the process [29]. Caffeine also reduces the impairment in cognitive and psychomotor tests and the anxiolytic effect of LOR [30].

Theoretical chemistry is important in identifying psychoactive controlled substances, especially for new psychoactive substances when only limited spectral data are available [31,32]. DFT is particularly useful for examining reaction mechanisms and intermediates [33]. The theoretical chemistry methods can be used for improved drug profiling by understanding the adulterants/diluents interactions with benzodiazepines and their effect on infrared and NMR spectra. These interactions also affect the stability and reactivity of benzodiazepines, and their identification and quantification are of utmost importance, especially in overdose and poisoning cases. Therefore, this paper aims to present the applicability of theoretical chemistry methods to examine benzodiazepine interactions with adulterants/diluents and vibrational spectra changes. The structures of lorazepam and alprazolam were optimized and compared to the experimental one to determine the appropriate level of theory. The experimentally obtained IR spectrum of lorazepam was reproduced following the optimization, and the experimental and theoretical wavenumbers were compared. The interactions between the benzodiazepines and adulterants/diluents (paracetamol, caffeine, glucose, and lactose) were investigated by optimizing structures and subjecting them to the quantum theory of atoms in molecules (QTAIM) analysis. The strongest interactions that could change the appearance of IR spectra of psychoactive substances are outlined.

## 2. Results 

### 2.1. Determination of the Appropriate Level of Theory

**Figure 1:**
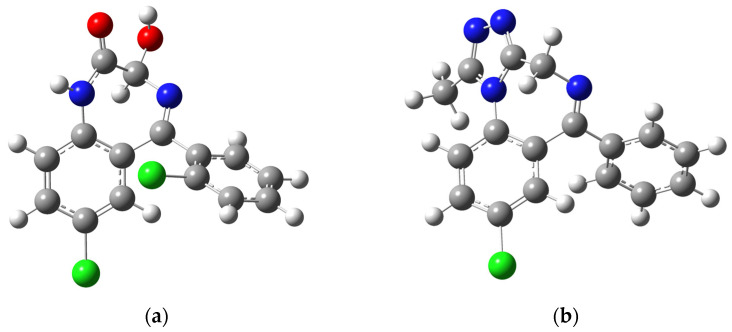
Optimized structures (at the M05-2X/6-311++G(d,p) level of theory) of (**a**) lorazepam and (**b**) alprazolam. (Hydrogen = white, carbon = gray, nitrogen = blue, oxygen = red, chlorine = green).

The crystal structures of alprazolam [34] and lorazepam [35] were optimized using several common functionals (B3LYP, B3LYP-D3BJ, CAM-B3LYP, B3PW91, M05-2X, and M06-2X) in conjunction with the 6-311++G(d,p) basis set. The optimized structures were compared to the experimental ones by calculating correlation coefficients and the mean absolute error (MAE). This parameter determines the average absolute difference between experimental and theoretical structural parameters. The values of bond lengths and angles are presented in Tables S1 and S2, while the MAE values are shown in Table 1. The optimized structures of ALP and LOR are depicted in Figure 1.

When experimental and theoretical bond lengths and angles were compared, it was concluded that all of the optimized structures resembled the experimental ones. The correlation coefficients for all investigated systems were 0.99 in the case of bond lengths and 0.99 and 0.95/0.96 for bond angles of ALP and LOR, respectively (Tables S1–S4). When LOR is concerned, the MAE values for bond lengths are in the narrow range between 0.013 (M05-2X) and 0.017 Å (B3LYP). On the other hand, the MAE values for bond angles are much more dispersed, between 0.91 (M05-2X) and 1.04° (B3LYP). The dispersion interactions in the functional B3LYP-D3BJ only slightly lowered the angle’s MAE value. Based on these values, it was concluded that the structure optimized at the M05-2X/6-311++G(d,p) level of theory was characterized by the lowest MAE values. The differences in bond lengths and angles between optimized and experimental structures of ALP were even less pronounced. The MAE values for bond lengths were of the order of experimental error (0.007–0.009 Å). The range of MAE values for bond angles was between 0.50 (M05-2X) and 0.59° (B3LYP). These results are consistent with the previous discussion; again, M05-2X showed the best result. Therefore, this functional was chosen for the further calculations.

The structures of LOR and ALP contain several structural elements that are important for the interactions with adulterants. Both compounds contain chlorine atom-attached aromatic rings. The natural bond orbital (NBO) charges of chlorine atoms are slightly positive, 0.034 (ALP) and 0.027/0.025 *e* (LOR), due to the electronegativity of aromatic rings. There are no significant differences in these charges depending on the other substituents and aromatic ring that these atoms are attached to in LOR. 1,4-diazepine rings contain two nitrogen atoms, both having negative charges. In the case of LOR, the nitrogen atom closer to the carbonyl group has a charge of −0.635 *e*, while the other has −0.434 *e*. The carbonyl oxygen atom is negatively charged (−0.621 *e*). The highest absolute value of negative charge was found for the hydroxyl group oxygen atom (−0.720 *e*). These four positions are suitable for hydrogen bond formation with the surrounding compounds, including adulterants and diluents. Two nitrogen atoms of a 1,4-diazepine ring of ALP have negative charges of −0.458 and −0.426 *e*. The similarity of these values is a consequence of the additional ring moiety in the structure of ALP. The substituted triazole ring moiety contains one of the mentioned nitrogen atoms and two additional nitrogen atoms with almost the same negative charge of around −0.3 *e*.

### 2.2. Comparison between Experimental and Theoretical ^13^C NMR and IR Spectra of Lorazepam

The ^13^C NMR and IR spectra of LOR were predicted and compared to the experimental ones as additional proof that the selected level of theory was applicable for their structural examination. The experimental ^13^C NMR spectra, recorded in DMSO, were taken from reference [36]. The structure of LOR was reoptimized at the M05-2X/6-311++G(d,p) level of theory in DMSO using the conductor-like polarizable continuum (CPCM) model to mimic the experimental condition. The chemical shifts were calculated using the gauge-independent atomic orbital (GIAO) method for the structure relative to TMS and the NMRdb predictor (www.nmrdb.org). The GIAO values were scaled by the linear fitting to the experimental values, as suggested in [16]. The scaling factor is necessary because the calculation was performed for the isolated molecule in a solvent polarizable continuum that does not include specific solvent–solute interactions. The values of chemical shifts are presented in Table S5 and compared by calculating the correlation coefficient and MAE value. The atom numeration is shown in Figure S1.

When experimental and theoretical ^13^C NMR chemical shifts were compared, it was concluded that both methods gave very high correlation coefficients (0.98 (GIAO) and 0.99 (NMRdb), Table S5). In the case of NMRdb, the MAE values were only 1.64 ppm, while with GIAO, the value was 2.63 ppm, consistent with the previous finding on ALP [16]. The lowest chemical shift was calculated for the sp^3^ hybridized carbon atom of the diazepine ring (83.1 (exp), 75.9 (GAIO), and 82.6 ppm (NMRdb)). The aromatic carbon atoms had chemical shifts between 123.4 and 137.4 ppm in the experimental spectrum and between 122.6 and 138.5 ppm in the GIAO predicted spectrum. The presence of a chlorine atom did not induce any significant change in the chemical shift. The presence of electronegative nitrogen atoms led to lower chemical shielding of carbon atoms of the diazepine ring, with high values of 162.3 (exp), 164.8 (GIAO), and 165.8 ppm (NMRdb) for the carbon atom in position 8. Additionally, the carbon atom of the carbonyl group had the highest values between 162.9 and 170.1 ppm for the experimental/theoretical methodologies. Nevertheless, an excellent general agreement between experimental and calculated chemical shifts should be outlined, which allows the use of the optimized structure for further analysis.

**Figure 2:**
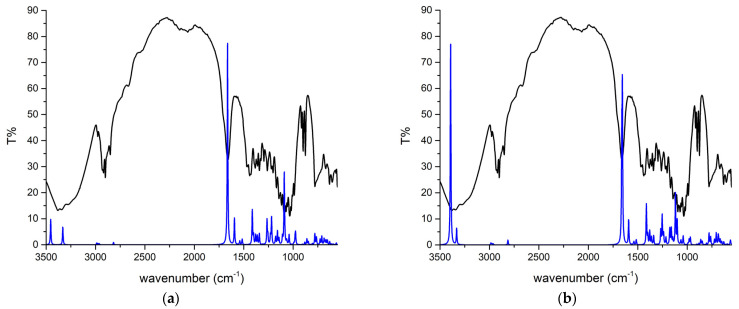
Experimental (black line) and theoretical (at M05-2X/6-31+G(d,p), blue line) IR spectra of (**a**) monomer and (**b**) dimer of lorazepam.

LOR was chosen because there were no theoretical analyses in which the experimental and theoretical spectra were compared at this level of theory. The theoretical structure was reoptimized at the M05-2X/6-31+G(d,p) level of theory that was later used to examine the interactions with adulterants/diluents due to the size of the system. The experimental IR spectrum of LOR was recorded after the extraction from commercial tablets. Because these spectra are commonly examined in daily forensic coursework, showing that theoretical spectra match the experimental ones is of utmost interest. The experimental and theoretical IR spectra of lorazepam are presented in Figure 2.

The theoretical wavenumbers were scaled by the factor of 0.910, determined using a least squares optimization computed to the experimental results. The same procedure was applied to compare experimental and theoretical spectra of various compounds [26,37,38]. The scaling factor was needed because the optimization was performed for the isolated molecule in a vacuum, while the experimental spectra were recorded for the solid sample in which the interactions of LOR with surrounding units can be expected. After recalculating the wavenumbers, the MAE value for comparison was 12 cm^−1^. As previously discussed, due to various electronegative atoms, these interactions lead to significant shifts in wavenumbers for the most pronounced bands, such as C=O, C=N, O–H, and C–H. The percentages of different vibrational modes in specific vibrations were determined by the potential energy distribution (PED) analysis in the VEDA program [39]. The experimental and scaled theoretical wavenumbers and the percentages of normal modes are listed in Table S6. It should be noted that additional bands might be present in the experimental spectrum, because the extraction of LOR was performed from the commercially available sample, and only those bands matching in experimental and theoretical spectra are discussed.

In the region between 3500 and 2000 cm^−1^, several bands are assigned to the X–H (X = O, N, H) stretching vibrations (Figure 2a). Due to interactions with the neighboring units, the O–H and N–H vibrations form a broad peak around 3400 cm^−1^ in both experimental and theoretical spectra. The theoretical position of the O–H stretching vibration is at 3454 cm^−1^, which is higher than the experimental one due to the lack of intermolecular interactions that lead to the elongation of the bond. The position of N–H vibrations varies significantly in organic compounds, between 3550 and 3300 cm^−1^, depending on the neighboring groups [40], and in LOR, it is assigned to the peak at 3335 cm^−1^ in experimental and 3331 cm^−1^ in theoretical spectra. This band is shifted toward lower wavenumbers in LOR due to partial delocalization within the diazepine ring. The phenyl ring C–H vibrations are partially covered by other bands in the experimental spectrum. The C–H group vibrations of the diazepine ring are positioned at 2846 and 2817 cm^−1^ in experimental and theoretical spectra, respectively, consistent with [41]. The most notable band in the IR spectrum is the C=O vibration at 1660 in the experimental and 1664 cm^−1^ in the theoretical spectrum, similar to other benzodiazepines containing this functional group [42]. This band is commonly used to identify LOR in seized samples in forensic coursework. The C=O vibration in the experimental partially covers the C=N vibration present at 1594 cm^−1^ in the theoretical spectrum. A similar position of this vibration was found for 4-hydroxyaprazolam in [43]. The C=C vibrations are assigned to bands at 1426 and 1493 cm^−1^ [44]. The intense band at 1080 and 1085 cm^−1^ in experimental and theoretical spectra belongs to the C–O stretching vibration. The C–N stretching vibration is predicted at 975 cm^−1^, which is in good agreement with the experimental band at 984 cm^−1^ [16]. Intense bands at 1259 (experimental) and 1255 cm^−1^ (theory) belong to the carbon–carbon stretching vibration, which includes carbon atoms connecting diazepine and phenyl rings [16]. The bands at 1036 and 1002 cm^−1^ are assigned to a complex motion with a significant contribution from the C–Cl vibration, as observed for alprazolam [16]. The rest of the bands correspond primarily to the C–C stretching and C–H bending and rocking modes. The band at 631 in the experimental and 654 cm^−1^ in the theoretical spectrum contains a significant contribution of C–Cl vibration. The most intense band, observed experimentally at 548 cm^−1^, includes several torsional and bending vibrations. The rest of the bands in this region contain deformational and torsional modes extended to all rings in the molecule, and they can be denoted as skeletal modes.

**Figure 3:**
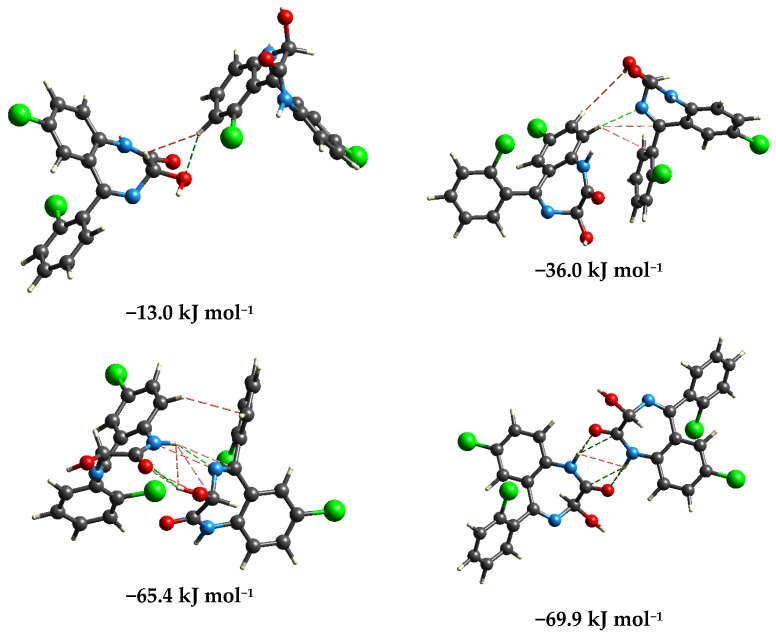
Structures with presented interactions within crystal structure (at the B3LYP/6-31G(d,p) level of theory, CrystalExplorer). (Hydrogen = white, carbon = gray, nitrogen = blue, oxygen = red, chlorine = green, green dashed lines represent hydrogen bonds, and red dashed lines represent close contacts).

The differences in experimental and theoretical IR wavenumbers result from different interactions within the solid LOR sample. These interactions were examined and quantified by the optimization at B3LYP/6-31G(d,p) in the CrystalExplorer program package, version 21 based on the mentioned crystal structure of LOR. Several dimer structures are presented in Figure 3, with the most important interactions denoted by green (hydrogen bonds) and red (closed contacts) dashed lines. The first dimer is characterized by a binding energy of −13.0 kJ mol^−1^, and it includes a weak hydrogen bond between the hydroxyl and C–H groups and weak interactions between the C–H groups. In the second dimer, the hydrogen bond is formed between the C–H groups and the nitrogen atom of the diazepine ring. Due to the lower distance between LOR molecules, other contacts exist between the C–H and hydroxyl/other C–H groups. These interactions lead to the stabilization of −36.0 C–H kJ mol^−1^. The third dimer has a significantly larger binding energy of −65.4 kJ mol^−1^. In this structure, one LOR molecule acts as a hydrogen atom donor (through the O–H group) and a hydrogen atom acceptor (through the diazepine nitrogen atom). Several weak interactions are formed between the electronegative and C–H groups. The most stable dimer structure is formed by the interactions between O–H and diazepine nitrogen of one LOR and carbonyl and the second diazepine nitrogen of the other LOR molecule (−69.9 kJ mol^−1^). As previously mentioned, these groups contain the most electronegative atoms within the structure of LOR, and these strong interactions can be expected in the prepared KBr pellet. These structures also show that groups in the most notable peaks in the IR spectrum readily interact with surrounding units, and shifts in the spectrum are due to the elongation of respective bonds.

To gain deeper insight into the type and strength of noncovalent interactions between two monomers, the structures of dimers were optimized at the M05-2X/6-31+G(d,p) level of theory and further examined by the QTAIM method, similar to [45]. According to this approach, the bond critical point (BCP) between two compounds or within one compound is characterized by the electron density (ρ(r)), Laplacian (∇^2^ρ(r)), Lagrangian kinetic electron density (*G*(r)), potential electron density (*V*(r)), density of total electron energy (*H*(r) = *G*(r) + *V*(r)), and interatomic bond energy (E_bond_ = *V*(r)/2), as discussed in reference [46]. Bader and Essen classified interactions as shared (covalent) with high electron density (>0.1 a.u.) and closed-shell (ionic bonds, van der Waals interactions, and hydrogen bonds) with an electron density of 0.01 a.u. [47].

**Figure 4:**
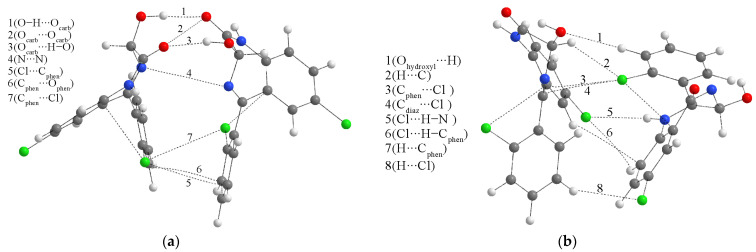
(**a**,**b**) QTAIM molecular graphs of the most stable dimer structures (at the M05-2X/6-31+G(d,p) of LOR. Dashed lines represent the bond paths. (Hydrogen—white, carbon—gray, nitrogen—blue, oxygen—red, chlorine—green).

The two dimers’ starting structures were prepared for the most common interactions from the previous section. Figure 4 presents optimized structures with specific interactions. The BCP parameters of these interactions are listed in Table S7. Upon inspection of critical points, it can be concluded that the Poincaré–Hopf relation is fulfilled for both structures. The binding energies, calculated by Equation (2), for dimers 1 and 2 are −44.9 and −35.8 kJ mol^−1^. At first sight, the structure of dimer 1 is characterized by two classical hydrogen bonds formed between hydroxyl and carbonyl groups. These interactions have an electron density of 0.024 a.u. and a Laplacian of 0.088 a.u., which puts them in the upper limit of the values proposed by Popelier and Koch for hydrogen bonding (0.002–0.040 a.u. (ρ(r)) and 0.024–0.139 a.u. (∇ ^2^ρ(r))). The ratio between −G(r) and V(r) is higher than 1, which indicates the purely noncovalent nature, additionally proven by the positive value of the total electron energy (3.6 kJ mol^−1^), as classified by Kremer and Kraka. These classical hydrogen bonds have the highest bond energy of −25.1 kJ mol^−1^, which has important implications for the experimental IR spectra. Any strong interaction formed by the carbonyl group will shift the peak used for identification. The theoretical band for the C=O vibration is shifted toward lower wavenumbers and widened due to these interactions (Figure 2b). The same theoretical spectrum contains the O–H stretching vibration band with increased intensity because of the elongation of the bond in the dimer structure. Interactions formed between electronegative elements, denoted as O_carb_∙∙∙O_carb_ and N∙∙∙N, are much weaker, with bond energies of −5.4 and −0.6 kJ mol^−1^. The chlorphenyl moieties also interact through weak interactions, such as Cl∙∙∙C_phen_ (−3.0 kJ mol^−1^), C_phen_∙∙∙C_phen_ (−2.7 kJ mol^−1^), and C_phen_∙∙∙Cl (−3.0 kJ mol^−1^). These interactions might also indicate the existence of π∙∙∙π contacts, as discussed in [45]. The bands of other vibrations are not significantly influenced by dimer formation.

On the other hand, the interatomic bond energies in the structure of the second dimer are much lower, between −1.1 and −6.9 kJ mol^−1^, with electron density, Laplacian, total bond energy, and −G(r)/V(r) indicating weak electrostatic–interatomic interactions. The strongest interactions within this dimer structure include chlorine and hydrogen/carbon atoms of phenyl and diazepine rings. The weak classical hydrogen bond, Cl∙∙∙H–N, has an interaction energy of −5.8 kJ mol^−1^. The wavenumbers of the theoretical C=O vibration and the intensities of O–H and N–H stretching vibrations are almost equal to those of the separate monomers. This analysis proves the assumption that the selection of relative positions of compounds in theoretical structures profoundly affects the quantitative parameters of interactions.

### 2.3. Molecular Dynamics Study of the Interactions of Lorazepam/Alprazolam with Adulterants/Diluents

Molecular dynamics simulations were employed to investigate the interactions between LOR/ALP with adulterants (caffeine and paracetamol) and diluents (glucose and lactose). An explicit model was used to examine these interactions, involving one LOR/ALP molecule surrounded by 100 adulterant/diluent molecules to mimic the tablets encountered at the crime scene. The system’s evolution, along with the formation of hydrogen bonds, was followed for five nanoseconds.

**Figure 5:**
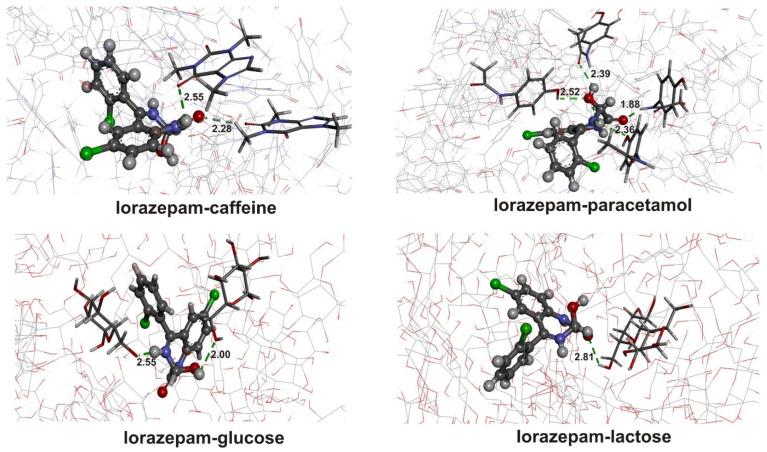
Possible interactions between lorazepam and adulterants/diluents. (Hydrogen—white, carbon—gray, nitrogen—blue, oxygen—red, chlorine—green, hydrogen bonds—green dashed line).

**Figure 6:**
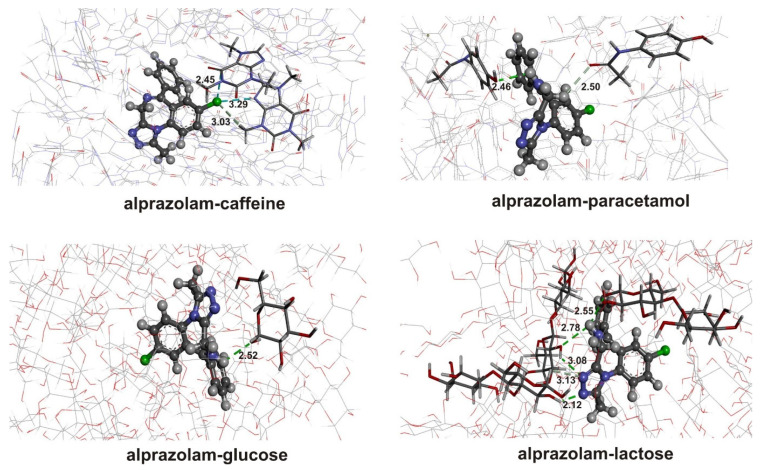
Possible interactions between alprazolam and adulterants/diluents. (Hydrogen—white, carbon—gray, nitrogen—blue, oxygen—red, chlorine—green, hydrogen bonds—green dashed line).

To quantify the interaction strengths, the average energies from all the simulation trajectories of LOR and ALP (E_L_ and E_A_), caffeine, paracetamol, glucose and lactose (E_C_, E_P_, E_G_, and E_Lac_), and complexes for each of the conjugates, E_L-C_, E_L-P_, E_L-G_, E_L-Lac_, E_A-C_, E_A-P_, and E_A-G_, E_A-Lac_ were taken. The system’s total energy is the sum of the potential and kinetic energies. The potential energy includes both bonded and nonbonded interactions within the system. Given that the presented study involves nonbonded interactions between the constituents, the discussion is focused on the nonbonded energy component along with the total energy to understand the system’s stability. The nonbonded energy is the sum of van der Waals and Coulombic energies. Three significant energy terms contributing to interactions in the system were considered: Coulombic, potential, and total energies. The interaction energy (ΔE) is calculated as the difference in energy of the conjugates (E_conjugates_) and the sum of the energies of the clusters (E_clusters_) and LOR/ALP (E_L-A_) [48]

(1) $${∆E}_{b}=E_{conjugates}-[E_{clusters}+E_{L/A}]$$

where E_clusters_ is the average energy of the clusters of 100 molecules of adulterants/diluents, and E_L-A_ is the average energy of LOR/ALP for their independent simulations. E_conjugates_ represent the energy of the LPR/ALP–adulterant/diluent conjugates in the MD simulations of the conjugated systems. The final structures are presented in Figure 5 and Figure 6, along with the energy contributions in Table 2. A negative change in average total energy (ΔE) indicates interactions and formation of stable conjugate of adulterants/diluents with LOR/ALP. When all conjugates containing LOR were compared, the strongest interactions were found between LOR and caffeine (−805.6 kJ mol^−1^). The final structure is characterized by the presence of two hydrogen bonds. The first is formed between the carbonyl group of caffeine and the protonated amino group of LOR (2.55 Å), while the second includes the methyl group of caffeine and carbonyl groups of LOR (2.28 Å). When LOR and paracetamol conjugates are examined, the total energy is lower (−554.0 kJ mol^−1^), although four hydrogen bonds were formed with distances between 1.88 and 2.52 Å. These interactions include amino/hydroxyl groups of paracetamol and carbonyl/hydroxyl groups of LOR. Hydroxyl groups present in structures of glucose and lactose additionally stabilize the system with LOR, −533.9 and −664.0 kJ mol^−1^, respectively. The higher stabilization of the system with lactose is expected due to the size of the molecule and the presence of other interactions, as shown later. It can be discussed that these hydrogen bonds are the main reason for the IR spectral changes of LOR in the presence of adulterants and diluents.

The structure of ALP is characterized by a higher number of electronegative atoms that could be included in forming hydrogen bonds, as previously explained. This is reflected in the molecular dynamics simulations through lower values of total energy compared to LOR, except in the case of conjugates with lactose. Three weak hydrogen bonds between the hydrogen atoms of caffeine and the chlorine atom of ALP were found, leading to the total energy of −823.1 kJ mol^−1^. The number of interactions is the main reason for lower total energy than LOR conjugates. In the case of paracetamol, weak hydrogen bonds are formed between carbonyl/hydroxyl groups of adulterant and hydrogen atoms of ALP. The total energy for this conjugate is lower for −264.6 kJ mol^−1^ than for LOR. Other weak hydrogen bonds are present in the conjugates with glucose and lactose. The evolution of the system, including lactose, leads to the structure with five hydrogen bonds with distances between 2.12 and 3.08 Å. Large distance values result from the diluent’s size and accommodation of the ALP molecule, which yields a lower total energy of −524.8 kJ mol^−1^. The Coulombic term is again the main contribution to the total binding energy. These preliminary results were further studied using the DFT method in the following section.

### 2.4. DFT/QTAIM Study of the Interactions of Lorazepam/Alprazolam with Adulterants/Diluents

The interactions with adulterants (paracetamol and caffeine) and diluents (glucose and lactose) were examined by optimizing three starting geometries with different positions of compounds from a previous study. These starting geometries were prepared in a way that included expected interactions. Only the most stable structures for each compound are discussed in the main text; for each pair of LOR with added compounds, the anticipated changes in IR spectra are discussed, along with the binding energy and QTAIM parameters of interactions. The optimizations were performed at the M05-2X/6-31+G(d,p) level of theory that is sufficiently high to examine the interactions due to the size of the system.

#### 2.4.1. Lorazepam and Paracetamol/Caffeine

Paracetamol consists of an aromatic ring with an attached hydroxyl group and an aliphatic chain with secondary amino and carbonyl groups. These polar groups are important for stabilization interactions with LOR once in the same formulation. Caffeine’s structure includes a higher number of electronegative atoms in two carbonyl groups and two adjacent ring structures with four nitrogen atoms in total.

**Figure 7:**
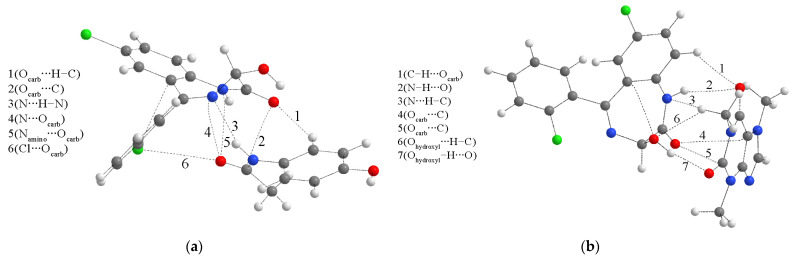
QTAIM molecular graphs of the most stable structures (at the M05-2X/6-31+G(d,p) level of theory) formed between LOR and (**a**) paracetamol and (**b**) caffeine. Dashed lines represent the bond paths. (Hydrogen—white, carbon—gray, nitrogen—blue, oxygen—red, chlorine—green).

The most stable structures formed between LOR and paracetamol/caffeine are presented in Figures S2 and S3. Different relative orientations between LOR and adulterants were critical to encountering different interactions. The binding energies in the case of paracetamol are −10.2, −39.6, and −43.3 kJ mol^−1^, indicating that the third structure is the most stable. When caffeine is concerned, binding energies are −33.1, −23.2, and −25.1 kJ mol^−1^. Based on these values, the strongest interactions are formed within the first structure. The QTAIM parameters for these structures are included in Figure 7 and Table S8 and further discussed in this section.

The stabilization interactions between LOR and paracetamol include several electronegative groups of both compounds. The strongest interaction is a hydrogen bond between the diazepine ring’s nitrogen atom and the paracetamol’s amino group, with an interatomic bond energy of −17.7 kJ mol^−1^. According to the Kramer and Kraka criteria, the positive total energy indicates weak and electrostatic interatomic interaction. The values of the electron density (0.019 a.u.) and the Laplacian (0.056 a.u.) fall within the range of values proposed by Popelier and Koch for hydrogen bonding. This interaction is also purely noncovalent, although the ratio between G(r) and V(r) is on the border proposed to discriminate covalent and noncovalent interactions. An unconventional hydrogen bond is also formed between the carbonyl oxygen of LOR and the C–H group of paracetamol with a bond energy of −8.6 kJ mol^−1^. The weaker character of this interaction is reflected in a higher ratio –G(r)/V(r), a lower electron density, and Laplacian. The interactions between electronegative atoms, denoted as N∙∙∙O_carb_, N_amino_∙∙∙O_carb_, and Cl∙∙∙O_carb_ have even lower bond energies of −6.7, −8.0, and −4.5 kJ mol^−1^. These energies are lower because the electronegativity of the included atom decreases. All of these interactions have positive total electron energy (1.1–1.3 kJ mol^−1^) and values of the ratio between G(r) and V(r) higher than 1, which classifies them as weak, noncovalent interactions. Additional interaction is found between LOR’s carbonyl oxygen and paracetamol’s carbonyl carbon atom, with a bond energy of −7.3 kJ mol^−1^.

The presence of two carbonyl groups in the structure of caffeine is important for the stabilization interactions. The strongest interaction between LOR and caffeine is denoted as O_hydroxyl_–H∙∙∙O, with a bond energy of −25.6 kJ mol^−1^. This interaction is characterized by high values of electron density (0.024 a.u.), Laplacian (0.076 a.u.), a negative total electron energy, and a ratio −G(r)/V(r) equal to 1. These parameters indicate the partial covalent character of this interaction, as discussed in [45]. This interaction also has some important indications in the spectral characteristics of this structure, as examined further. An unconventional hydrogen bond is also formed between the hydroxyl oxygen atom of LOR and the C–H bond with an energy of −9.7 kJ mol^−1^, with other parameters classifying it as a noncovalent interaction. The carbonyl oxygen of LOR is included in two interactions with carbon atoms with energies of −9.1 and −6.6 kJ mol^−1^. These interactions have electron densities around 0.010 a.u., Laplacian around 0.030 a.u., and positive total electron energies. The phenyl ring C–H group interacts with the second carbonyl oxygen of caffeine with a bond energy of −5.0 kJ mol^−1^ through a purely noncovalent interaction. The amino group of LOR is included in interactions with carbonyl oxygen (N–H∙∙∙O) and carbon atoms (N∙∙∙H–C) with interaction energies of −9.0 and−6.1 kJ mol^−1^.

**Figure 8:**
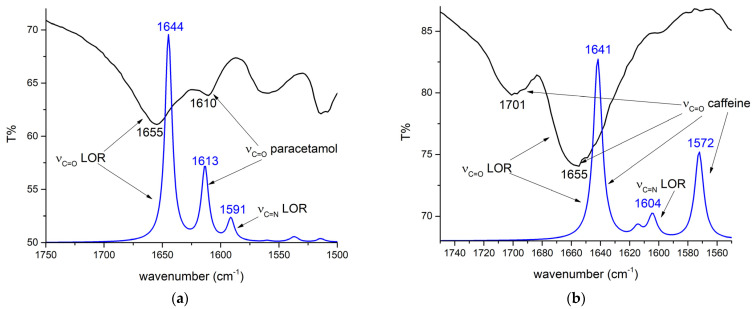
Experimental (black line) and theoretical (at the M05-2X/6-31+G(d,p) level of theory, blue line) IR spectra of LOR extract with (**a**) paracetamol and (**b**) caffeine.

The mentioned interactions with adulterants lead to specific changes in IR spectra. The experimental IR spectra of LOR and paracetamol/caffeine were compared to the calculated ones at the M05-2X/6-31+G(d,p) level of theory. The previously computed correction factor was applied to calculate theoretical wavenumbers. As previously explained, the carbonyl group stretching vibration of LOR is one of the most important bands, usually used to verify the presence of LOR in seized samples. Therefore, the discussion in this section will mainly include changes in the position of this band. When paracetamol is mixed with LOR, there is a slight change in the position of the C=O stretching band to 1655 cm^−1^, which is 5 cm^−1^ lower than in the pure LOR sample, as shown in Figure 8a. This band is shifted to 1644 cm^−1^ in the theoretical spectra due to the aromatic ring’s interactions with hydrogen and carbon atoms through weak hydrogen bonds (Figure 8a). In the experimental spectrum, an additional band at 1610 cm^−1^ is attributed to the C=O stretching vibration of paracetamol. The position of this band is well reproduced in the theoretical spectrum (1613 cm^−1^). This result proves the assumption that the addition of adulterants might lead to changes in the positions and the relative intensity of the bands of psychoactive substances, although in the case of the LOR–paracetamol pair, there is no overlap between bands. The analysis of experimental IR spectra when caffeine is present is much more complex, because there is a new band at 1701 cm^−1^, while the band’s intensity at 1655 cm^−1^ is significantly increased (Figure 8b). This indicates a significant overlap between bands belonging to caffeine and LOR, which limits the identification and quantification of present LOR in samples. The position of the carbonyl stretching vibration of LOR is shifted because of the interactions with the aromatic rings of caffeine. In the theoretical spectrum, the C=O stretching bands of LOR and caffeine overlap at 1641 cm^−1^, and this change explains the experimentally observed increase in intensity of this band. The second carbonyl group vibration of caffeine is also located at 1572 cm^−1^. The differences in positions in theoretical and experimental IR spectra are due to the significant susceptibility of the carbonyl group to the formation of interactions with surrounding units, which limits the exact calculation of their position. Nevertheless, this analysis proved that the addition of adulterants has a two-fold purpose: one is to enhance the activity of active substances, and the other one is to cover the most important bands in the IR spectrum. Introducing more adulterant molecules would produce a better match in the wavenumber values.

#### 2.4.2. Lorazepam and Glucose/Lactose

**Figure 9:**
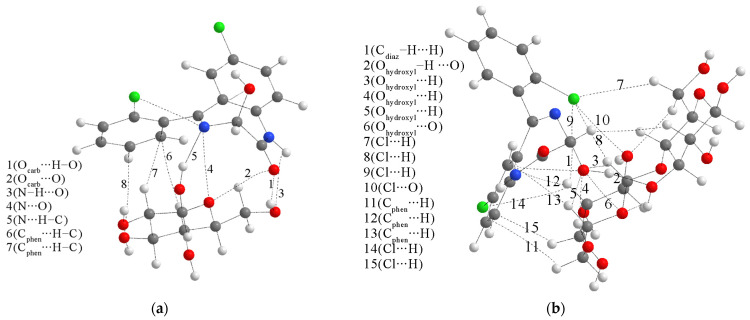
QTAIM molecular graphs of the most stable structures (at the M05-2X/6-31+G(d,p) level of theory) formed between LOR and (**a**) glucose and (**b**) lactose. Dashed lines represent the bond paths. (Hydrogen—white, carbon—gray, nitrogen—blue, oxygen—red, chlorine—green).

Glucose and lactose are two common sugars added to increase the bulk substance. These two compounds contain multiple hydroxyl groups that are included in forming hydrogen bonds with LOR, as examined in the following section. The most stable structures formed between LOR and glucose/lactose are presented in Figures S4 and S5. When binding energies are compared for the structures formed between LOR and glucose (−37.7 (structure 1), −19.8 (structure 2), and −34.4 kJ mol^−1^ (structure 3)), the structure chosen for further examination is structure 1. The same type of analysis was performed for structures obtained in LOR–lactose pairs. The binding energies are −43.1, −21.6, and−50.9 kJ mol^−1^ for structures 1–3. The strongest interactions are found in structure 3. The QTAIM plots showing the most important stabilization interactions are shown in Figure 9.

As expected, the interactions between glucose and LOR are higher than previously discussed for paracetamol and caffeine. There are two partially covalent interactions denoted as O_carb_∙∙∙H–O (−23.2 kJ mol^−1^) and N∙∙∙H–C (−28.6 kJ mol^−1^) with negative total energies of −1.4 and −0.7 kJ mol^−1^, respectively, and −G(r) values equal to 1. Another conventional hydrogen bond is formed between the protonated nitrogen of LOR and oxygen of glucose, with a bond energy of −11.7 kJ mol^−1^. This interaction is noncovalent, with H(r) > 1 and −G(r)/V(r) > 1. Due to the abundance of oxygen atoms in the structure of glucose, three interactions include a direct interaction between oxygen (O_carb_∙∙∙O), nitrogen (N∙∙∙O), phenyl carbon (C_phen_∙∙∙O) atoms with bond energies between −1.9 and −7.5 kJ mol^−1^. The energies of these interactions increase with the electronegativity of atoms. These three interactions can be classified as weak, with an electron density between 0.003 and 0.008 a.u. The interactions between phenyl carbon and C–H bonds of glucose present the alkyl-π interactions between two compounds. Their energies are −5.1 and −3.0 kJ mol^−1^ with positive total energy and a ratio between G(r) and V(r) equal to 1.2 and 1.4.

The number of interactions significantly increases when lactose is added to the samples due to the increased number of hydroxyl groups. The strongest interaction, with the interatomic bond energy −46.5 kJ mol^−1^, is formed between the hydroxyl group of LOR and the oxygen atom of lactose. When energies of all mentioned interactions are compared, this one was the highest. The partial covalent character of this interaction is shown by the negative value of total electron energy (−2.2 kJ mol^−1^) and the −G(r)/V(r) value equal to 1. The same oxygen atom of LOR is included in three other interactions with neighboring hydrogen atoms, with bond energies of −13.2, −8.8, and −5.1 kJ mol^−1^, and one oxygen atom of lactose (−7.5 kJ mol^−1^). All of these interactions are noncovalent, with −G(r)/V(r) > 1.0. Two chlorine atoms of LOR form interactions with the hydrogen and oxygen atoms of lactose. These interactions are characterized by much lower interaction energies, between 1.6 and 4.2 kJ mol^−1^. Electron densities and Laplacian values are also lower compared to the parameters for interactions, including oxygen atoms. The phenyl carbon atoms form interactions with the hydrogen atoms of lactose, with interaction energy that depends on the distance and proximity of electronegative elements. As previously mentioned, these interactions are present between two rings and additionally stabilize structures. The weakest interaction is the one found between the hydrogen atoms of the diazepine ring and lactose, with an interaction energy of only −0.8 kJ mol^−1^.

**Figure 10:**
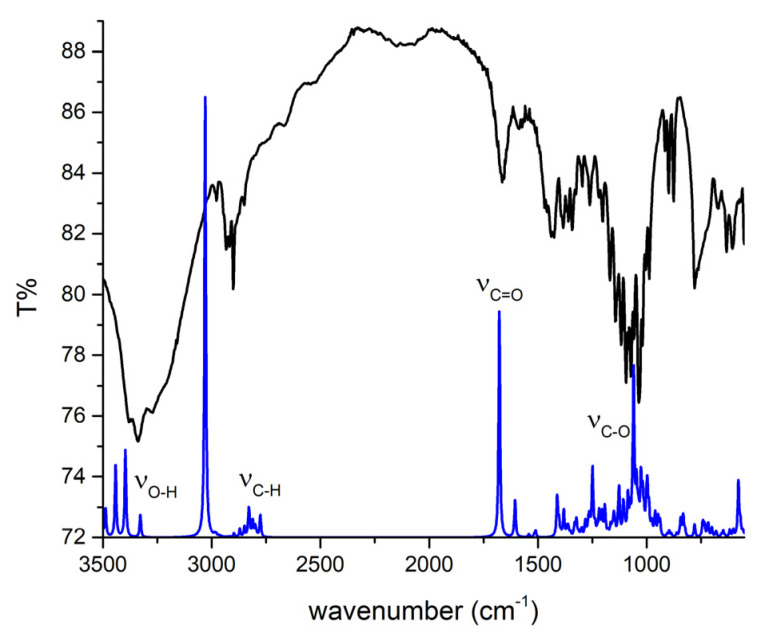
Experimental (black line) and theoretical (at the M05-2X/6-31+G(d,p) level of theory, blue line) IR spectra of the LOR tablet with lactose.

The experimental IR spectrum of LOR tablets with lactose as the diluent is compared to the theoretical spectrum of the LOR–lactose structure in Figure 10. Again, the theoretical wavenumbers were corrected by the same correction factor. In the experimental spectrum, the band belonging to the carbonyl stretching vibration has a much lower intensity than that observed in Figure 2 due to a low concentration. The bands belonging to the O–H and C–O stretching vibrations of lactose are much more pronounced. The position of the C=O stretching vibration is almost identical to the pure LOR sample (1662 cm^−1^) in the experimental and theoretical (1667 cm^−1^) spectrum. It can be assumed that the interactions with lactose influence the whole structure of LOR due to the size of the diluent’s molecule. This is especially evident in the intensity of the O–H stretching vibration of LOR due to strong interaction with hydroxyl oxygen atoms, as discussed in the previous paragraph (Table S7). The positions of C–H and C–O vibrations of lactose are also present in their experimentally observed positions. There is a high resemblance between experimental and theoretical spectra, which proves that theoretical chemistry calculations can predict spectra of complex mixtures if the constituents are known and their relative positions are determined well.

#### 2.4.3. Alprazolam and Paracetamol/Caffeine

**Figure 11:**
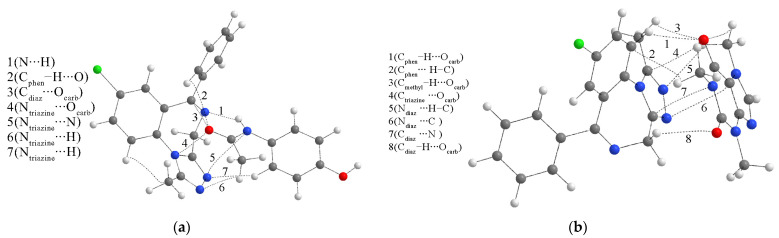
QTAIM molecular graphs of the most stable structures (at the M05-2X/6-31+G(d,p) level of theory) formed between ALP and (**a**) paracetamol and (**b**) caffeine. Dashed lines represent the bond paths. (Hydrogen—white, carbon—gray, nitrogen—blue, oxygen—red, chlorine—green).

The structures containing ALP and paracetamol were prepared by positioning two interacting molecules to include all polar group interactions. The optimized structures are presented in Figure S6, and the following binding energies are calculated: −35.9 (structure 1), −42.9 (structure 2), and −65.6 kJ mol^−1^ (structure 3). Structure 3 was chosen for further analysis, and the most important interactions are given in the following figure. The interactions with caffeine were also examined with binding energies of −58.1, −11.8, and −42.7 kJ mol^−1^ for structures 1–3, respectively (Figure S7). The QTAIM parameters of the most stable structures are shown in Table S9 and Figure 11.

When binding energies between ALP and paracetamol are compared to the same pairs with LOR, it can be observed that they are higher for 10–15 kJ mol^−1^, which is consistent with the molecular dynamic simulations. Careful inspection based on the QTAIM parameters allows justification for this finding. As presented in Table S9, the strongest interaction between ALP and paracetamol is the conventional hydrogen bond N∙∙∙H–N, with an electron density of 0.014 a.u. and a Laplacian of 0.041 a.u, in which ALP acts as a hydrogen atom acceptor. The interatomic bond energy is −12.3 kJ mol^−1^. Two other interactions are found between carbon atoms of diazepine, phenol rings, and carbonyl oxygen, with energies of −7.0 and −8.4 kJ mol^−1^. An important group of interactions between ALP and paracetamol includes triazine nitrogen atoms. These interactions, with energy between −4.1 (N_triazine_∙∙∙H) and −12.3 kJ mol^−1^ (N_triazine_∙∙∙O_carb_), are the probable reason for the higher stability of the structures formed between ALP and paracetamol than those between LOR and paracetamol. The triazepine ring with three two additional nitrogen atoms pointing in the same direction is much more involved in the interaction formation than the carbonyl and hydroxyl groups of LOR. Therefore, it can be concluded that although the separate interactions are less strong, their number significantly influences the overall stability.

The previous finding is much more pronounced in the interaction between ALP and caffeine. The most stable structure between ALP and caffeine has an interaction energy of −58.1 kJ mol^−1^, 25 kJ mol^−1^ lower than the interaction energy between LOR and caffeine. The strongest interaction is a weak hydrogen bond denoted as C_phen_–H∙∙∙O_carb_ with an interaction energy of −10.0 kJ mol^−1^. Two additional weak hydrogen bonds of the same type have energies of −5.2 and 4.7 kJ mol^−1^ (Table S9). The diazepine nitrogen atoms interact with carbon and hydrogen atoms through weak noncovalent interactions. The carbon atoms of ALP also form interactions with carbonyl oxygen, nitrogen, and hydrogen atoms. The absence of a hydroxyl group in ALP is responsible for lower separate energies of interactions. Still, the presence of several electronegative atoms leads to a higher number of interactions, which gives a higher absolute value of binding energy.

#### 2.4.4. Alprazolam and Glucose/Lactose

**Figure 12:**
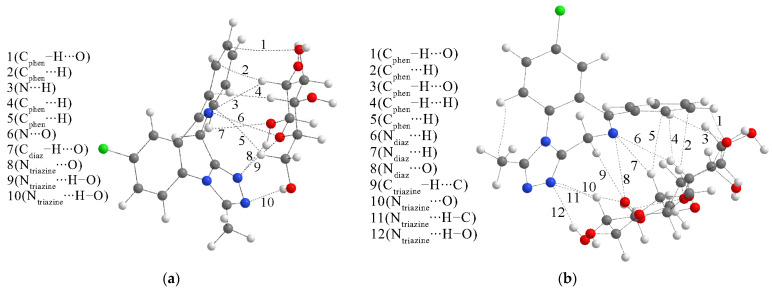
QTAIM molecular graphs of the most stable structures (at the M05-2X/6-31+G(d,p) level of theory) formed between ALP and (**a**) glucose and (**b**) lactose. Dashed lines represent the bond paths. (Hydrogen—white, carbon—gray, nitrogen—blue, oxygen—red, chlorine—green).

The interactions between ALP and glucose led to the formation of three structures with stabilization energies of −66.0 (structure 1), −34.9 (structure 2), and −42.2 kJ mol^−1^ (structure 3) (Figure S8). Based on these values, structure 1 was selected for further investigation of the QTAIM parameters and possible changes in IR spectra. The stabilization energies for the interactions with lactose were more uniform, between −37.8 (structure 1) and −42.6 kJ mol^−1^ (structure 3), as depicted in Figure S9. The most stable structures and QTAIM interactions are shown in Figure 12.

The interactions between ALP and glucose include two hydrogen bonds between triazine nitrogen atoms and hydroxyl groups with bond energies of −23.1 and −11.0 kJ mol^−1^, with the partial covalent character as the −G(r)/V(r) value equal to 1 (Table S9). There are two additional stabilization interactions between the nitrogen atoms of ALP and oxygen atoms of glucose with bond energies of −9.4 and −4.9 kJ mol^−1^ that can be classified as purely noncovalent interactions with positive values of total electron energy (1.3 and 1.9 kJ mol^−1^). There are several interactions between phenyl and diazepine carbon and hydrogen atoms of ALP and hydrogen and oxygen atoms of glucose. These interactions are characterized by electron densities between 0.004 and 0.007 a.u. and Laplacian values between 0.012 and 0.026 a.u. These weak interactions are more numerous than in the case of LOR, leading to higher stabilization of around 30 kJ mol^−1^. The relative positions of triazepine and phenyl rings of ALP allow a higher number of interactions with the glucose molecule.

Similar was found for the interactions between ALP and lactose. The strongest interaction with a bond energy of −15.4 kJ mol^−1^ is denoted as N_triazine_∙∙∙H–O. Triazine nitrogen atoms are included in another weak hydrogen bond (N_triazine_∙∙∙H–C) and interaction with the oxygen atom (N_triazine_∙∙∙H–O). These interactions have bond energies of −6.7 and −6.0 kJ mol^−1^. The diazepine nitrogen atom forms three interactions with hydrogen and oxygen atoms of lactose with relatively low values of electron density and Laplacian. Due to the size of the lactose molecule and its alignment toward diazepine and phenyl rings, several interactions between phenyl carbon atoms and oxygen/hydrogen are found. Two weak hydrogen bonds in which ALP acts as a hydrogen atom donor have bond energies of −5.2 and −4.4 kJ mol^−1^. In general, stabilization energies between ALP and lactose are higher than those calculated for LOR and lactose. However, the most stable structure with LOR is more stable than that with ALP. The reason is the orientation of LOR that allows the formation of a strong hydrogen bond with a bond energy of −46.5 kJ mol^−1^, as previously discussed.

## 3. Discussion

Adulteration and dilution of solid samples are commonly encountered in forensic cases [49]. These compounds are added to increase the volume of samples and enhance some of the effects. The theoretical chemistry methods are helpful for predicting spectral changes and analyzing interactions due to the mixing of psychoactive compounds with adulterants and diluents. Before using quantum chemical calculations, the appropriate level of theory should be determined by comparing the optimized bond lengths and angles with the crystallographic ones of LOR and ALP. In this contribution, five common functionals were selected in conjunction with the 6-311++G(d,p) basis set. The lowest MAE and highest R values were obtained for the M05-2X/6-311++G(d,p) level of theory. The selected functional is recommended for examining the noncovalent interactions, which is important for further quantum chemical assessment of the interactions between benzodiazepines and adulterants/diluents [50,51]. Although structurally similar, substituents and their relative position in structures of LOR and ALP could potentially lead to pronounced differences in interactions with adulterants and diluents.

The applicability of the chosen level of theory was verified by the prediction and assignation of vibrational and ^13^C NMR spectra of LOR. ALP’s assigned theoretical and experimental spectra are already shown in reference [16]. A high correlation coefficient (0.98) and low MAE value (2.63 ppm) proved that the selected level of theory was appropriate for predicting ^13^C NMR spectra of LOR. The scaling factor was obtained to compare experimental and theoretical IR spectra of LOR. The scaling factor did not alter the positions of peaks but allowed better assignment of some close-lying bands [52]. The MAE value for the comparison of vibrational spectra was 12 cm^−1^. These differences result from the physical state of the sample used for FTIR measurements, anharmonicity effect, and various approximations of the theoretical chemistry methods, as explained for amphetamine and its derivatives [53].

The differences in experimental and theoretical wavenumbers are a consequence of the interactions within the crystal structure of LOR. The interactions’ energy was determined in the CrystalExprorer at the B3LYP/6-31G(d,p) level of theory. This level of theory proved sufficient for predicting trends in interaction strengths, as shown in [54,55,56]. Dimer structures were characterized by the interactions that influence the wavenumbers of the most prominent bands in the IR spectrum. The most stable dimer included interactions between two diazepine rings through the carbonyl groups, which is important for understanding the necessity of the obtained scaling factor. The considerations of intramolecular interactions have significant implications in examining the vibrational spectra of novel psychoactive substances, because discrepancies between experimental and theoretical wavenumbers are usually encountered [57]. Additionally, quantification of the interactions’ strength was performed by the QTAIM analysis. This approach proved useful for examining the electronic structure of molecules and intra-/intermolecular interactions of amphetamines [53] and benzodiazepines [45] in other theoretical analyses.

Theoretical chemistry methods offer a possibility for investigating the interactions between two isolated compounds, a crucial step for further discussion of the adulterants/diluents’ effects on the stability and spectra of psychoactive compounds. These might include the interactions between NPS and biomolecular targets [58,59] and other active compounds or solvent molecules [60]. Molecular dynamics was applied to examine the relative positions of benzodiazepines and adulterants/diluents. These interactions were quantified by calculating the interaction energy, as proposed in [48]. Due to the higher number of electronegative atoms, the interactions with ALP were stronger in almost all cases except for those with lactose. The main contribution to these interactions was the Coulombic term.

The optimization of several benzodiazepine–adulterant/diluent pairs was performed by selecting four different structures from molecular dynamics simulations. Previously, the same procedure was applied to investigate interactions between serotonin and solvent molecules [61] and escitalopram and ibuprofen/paracetamol [62]. These calculations are especially important when UV–VIS spectral characteristics of NPS in solutions are examined [61]. Only the most stable structures, including ALP and LOR, out of four pairs, underwent QTAIM analysis. The strongest interactions between LOR and paracetamol were the hydrogen bonds, characterized by the QTAIM parameters proposed by Popelier and Koch [63]. These interactions induced a shift in carbonyl group vibration of LOR to 1655 cm^−1^ in experimental and 1644 cm^−1^ in theoretical spectra. This result verifies the assumption that the presence of different adulterants could affect IR spectral bands in solid samples. The importance of this finding is in the fact that theoretical chemistry methods could be applied for the examination of the complex mixtures of NPS and adulterants if their chemical nature is known. On the other hand, in the presence of caffeine, the most important band in LOR’s IR spectrum is covered by the band of the caffeine carbonyl group. Other than the shift in position, the change in intensity of bands can be explained by the overlap of the bands. As previously discussed, appropriate determination of structural parameters is essential for predicting vibrational frequencies. Commonly encountered adulterants could be optimized, and their behavior in the presence of different NPS could be examined. This would be an initial step for the spectral analysis of mixtures found at the crime scene. Diluents, such as glucose and lactose, usually have several hydroxyl groups that readily interact with electronegative groups of NPS but do not cover the important bands. The theoretical spectrum of the LOR–paracetamol mixture well-reproduced the experimental one, proving that the selection of pair NPS/diluents can be used for the IR spectral prediction. LOR’s carbonyl peak position was unchanged in the mixture (commercially available tablet) and the pure sample.

The interactions between ALP and paracetamol/caffeine were stronger than those with LOR, consistent with the molecular dynamic simulations. Because ALP does not contain a carbonyl group, other bands are important for vibrational spectroscopy identification. The presence of adulterants affects the vibrations of ALP’s triazine ring through the formation of hydrogen bonds between electronegative groups. The addition of diluents also influences the spectra by the formation of hydrogen bonds between the nitrogen atoms of the ALP and hydroxyl groups.

The methodology presented in the contribution is a pivotal step toward applying theoretical chemistry methods in examining the complex mixtures commonly found at the crime scene. The appropriate conformational and structural analysis and comparison with the spectra of pure substances (if available) is crucial for the further investigation of the interactions. If the crystal structure of NPS is unknown, the optimization and comparison can be performed with structural analogs and applied to the structure prediction. This later allows the calculation of vibrational, NMR, and mass spectra of compounds and their identification in samples upon separation. Changes in spectra can be observed in mixtures of NPS with adulterants/diluents. They can be quantified by the DFT optimization and subsequent QTAIM analysis and vibrational/NMR spectra prediction. This approach would have significant implications in the ever-growing field of NPS.

## 4. Materials and Methods

### 4.1. Chemicals

Paracetamol, caffeine, ethanol, and potassium bromide were obtained from Merck & Co. (Rahway, NJ, USA) as p.a. chemicals and used without purification.

### 4.2. Extraction of Lorazepam from Comercial Tablets

A Fourier-transform infrared (FTIR) spectrophotometric method was developed to measure lorazepam in ethanol extract rapidly. For this purpose, ethanol extraction was used to extract lorazepam from 2.5 mg lorazepam tablets as a commercial product (Lorazepam HF (Hemofarm, Vršac, Serbia)). A portion of 10 mL of ethanol is sufficient for extraction from four tablets. After that, the extract was filtered, dried, and heated at 70 °C until dry. The extract was recorded on an Thermo Nicolet Avatar 370 FTIR spectrometer (Thermo Fisher Scientific, Waltham, MA, USA) in the 3500–500 cm^−1^ range. The average spectra from 32 scans were included in this publication. The spectra of lorazepam with paracetamol/caffeine were obtained for their mixture, miming the common ratio between active compounds and adulterants in samples.

After the extraction of lorazepam, paracetamol (0.040 mg) was added. The spectrum recorded by FTIR showed that paracetamol partially covered the spectrum of lorazepam, but the main wavelengths of paracetamol were still observed.

Then, 0.025 mg of caffeine was added to the lorazepam extract, a lower amount that was enough to completely cover the lorazepam spectrum.

### 4.3. Theoretical Analysis

The crystal structures of lorazepam and alprazolam were taken from the Cambridge Crystallographic Data Center (CCDC) as entries 1131103 [64] and 139757 [34], respectively. These structures were optimized in the Gaussian 09 Program package [65] by employing several common functionals (B3LYP, CAM-B3LYP, B3PW91, M05-2X, and M06-2X) [66,67,68,69,70,71] in conjunction with the 6-311++G(d,p) [72] level of theory. The dispersion interactions were included in the case of the B3LYP-D3BJ functional [73]. The optimizations were performed without any geometrical constraints, and the absence of imaginary frequencies identified the structures corresponding to the local minima. Once the appropriate level of theory (M05-2X/6-311++G(d,p)) was determined, the structures of caffeine, paracetamol, glucose, and lactose were optimized. For both benzodiazepines, three of the most stable conformations with each adulterant (24 in total) were selected and optimized at the M05-2X/6-31+(d,p) level of theory. The vibrational frequencies were determined to verify that minima on the electronic energy surface were found. The vibrations were visualized in the GausView program package [74]. The natural changes were determined through the natural bond orbital (NBO) theory approach [75,76]. The ^13^C NMR spectra of LOR were predicted using the NMRBD spectral predictor (www.nmrdb.org, 15 July 2024) [77,78]. The theoretical ^13^C NMR chemical shieldings were calculated by the gauge-independent atomic orbital (GAIO) approach [79,80] at the M05-2X/6-311++G(d,p) level of theory. The chemical shieldings were converted to chemical shifts by subtracting values from the tetramethylsilane (TMS) chemical shielding optimized at the same theory level. These calculations were performed on the structures optimized in DMSO to mimic the experimental conditions using the conductor-like polarizable continuum (CPCM) model [81].

The binding energy (E_b_) was determined by the following equation, as suggested by Parlak and coworkers [62]:

(2) $$E_{b}=E_{complex}-[E_{benzodiazepine}+E_{adulterant}]$$

where E_complex_, E_benzodiazepine_, and E_adulterant_ are the optimized energies of the complex between the benzodiazepine and adulterant, benzodiazepine, and adulterant, respectively. The basis set superposition errors (BSSEs) were included by the counterpoise correction method. The interaction energies within the crystal structure of LOR were determined in the CrystalExplorer program [69] at the B3LYP/6-31G level of theory.

The quantum theory of atoms in molecules (QTAIM), proposed by Bader [82,83], was applied to investigate different interactions in optimized structures. The .wfx files from Gaussian 09 were subjected to further calculations in the AIMAll program [84].

### 4.4. Molecular Dynamics Simulations

Molecular dynamics (MD) simulations were performed with the GROMACS software package, version 2024.2 [85]. All systems were simulated with a DREIDING force field [86]. The energetic minimization of the neutralized system was performed by the steepest descent and conjugate gradient algorithms with up to a tolerance of 1000 kJ mol^−1^ nm^−1^ during 5000 steps. After that, the system was equilibrated at 310.15 K using the Berendsen weak coupling method in the NVT (constant volume) ensemble condition with a 2 ns time scale. The LINCS algorithm was used for the production of the MD phase in the NPT ensemble for a 5 ns time scale, including a modified Berendsen thermostat (τT = 1 ps) and a Parrinello–Rahman barostat (τP = 2 ps) [87].

## 5. Conclusions

The LOR and ALP crystal structures were optimized using several common functionals in conjunction with 6-311++G(d,p). The lowest mean absolute errors for comparing experimental and theoretical bond lengths and angles were obtained for the structure optimized at M05-2X/6-311++G(d,p). The ^13^C NMR spectra of LOR were calculated using the GIAO method, which gave a correlation coefficient of 0.98 and MAE of 2.63 ppm. The experimental positions of LOR’s most prominent bands in the IR spectrum were well reproduced, with an MAE value of 12 cm^−1^. The differences in experimental and theoretical IR spectra were explained by intramolecular interactions between LOR monomers. Several dimer structures were obtained from the crystal structure, and stabilization energies were calculated at B3LYP/6-31G(d,p). These interactions include the polar groups of LOR, which led to changes in the theoretical spectrum of isolated substances. These interactions were further examined using the QTAIM approach for two dimer structures. Molecular dynamic simulations were performed for a system consisting of 1 benzodiazepine molecule and 100 molecules of adulterants/diluents. The total energy of interaction was highest for the interactions between lorazepam and alprazolam with caffeine, −805.6 and −823.1 kJ mol^−1^, respectively. Hydrogen bonds formed between compounds were listed. The most stable structures were optimized at the M05-2X/6-31+G(d,p) level of theory, and QTAIM parameters were calculated. The changes in spectra of LOR upon the addition of paracetamol, caffeine, and lactose were explained by the formed interactions. The stabilization of the system was more pronounced in the case of ALP due to the presence of a higher number of electronegative atoms. The presented methodology is a crucial advancement in applying theoretical chemistry to analyze complex mixtures, including controlled substances often found at crime scenes. This approach enables the prediction of vibrational spectra, aiding in identifying compounds within mixtures and offering valuable insights into controlled substances and their interactions with adulterants or diluents.

## Figures and Tables

**Table 1:** Mean absolute error for comparing experimental and theoretical bond lengths and angles of alprazolam and lorazepam.

Compound		B3LYP	B3LYP-D3BJ	B3PW91	CAM-B3LYP	M05-2X	M06-2X
Lorazepam	Bond lengths	0.017	0.015	0.014	0.014	0.013	0.014
	Bond angles	1.04	0.95	1.02	0.99	0.91	0.92
Alprazolam	Bond lengths	0.009	0.009	0.008	0.007	0.007	0.008
	Bond angles	0.59	0.52	0.56	0.58	0.50	0.52

**Table 2:** The interaction energy of the simulated systems.

ΔE (kJ mol^−1^)			
	Total Energy	Potential Energy	Coulombic
lorazepam–caffeine	−805.6	−689.4	−366.8
lorazepam–paracetamol	−554.0	−437.5	−128.3
lorazepam–glucose	−533.9	−418.0	−244.1
lorazepam–lactose	−664.0	−547.4	−366.8
alprazolam–caffeine	−823.1	−698.6	−272.6
alprazolam–paracetamol	−818.6	−693.4	−38.23
alprazolam–glucose	−723.9	−599.1	−92.61
alprazolam–lactose	−524.8	−399.6	−54.22

## Data Availability

Data are contained within this article.

## Supplementary Materials

The following supporting information can be downloaded at: https://www.mdpi.com/article/10.3390/ijms251810087/s1.
