# Supplementary material for: Molecular Aspects of the Interactions between Selected Benzodiazepines and Common Adulterants/Diluents: Forensic Application of Theoretical Chemistry Methods
Source: Int J Mol Sci. 2024 Sep 19;25(18):10087. doi: 10.3390/ijms251810087 (PMC11432270; doi:10.3390/ijms251810087)
Supplement: Supplementary file 1 [file ijms-25-10087-s001.zip › ijms-3169321-supplementary.pdf]

Supplementary information for:

Molecular Aspects of the Interactions between Selected Benzodiazepines and Common Adulterants/Diluents: Forensic Application of Theoretical Chemistry Methods

Jelica Džodić [1], Milica Marković [2], Dejan Milenković [2] and Dušan Dimić [2,*]

[1] Faculty of Physical Chemistry, University of Belgrade, Studentski trg 12-16, 11000 Belgrade, Serbia; jelica.dzodic.993@gmail.com (J.Dž.); milica.markovic@ffh.bg.ac.rs (M.M.); ddimic@ffh.bg.ac.rs (D.D.)

[2] Department of Science, Institute for Information Technologies, University of Kragujevac, Jovana Cvijića bb, 34000 Kragujevac, Serbia; dejanm@uni.kg.ac.rs (D.M.)

* Correspondence: ddimic@ffh.bg.ac.rs

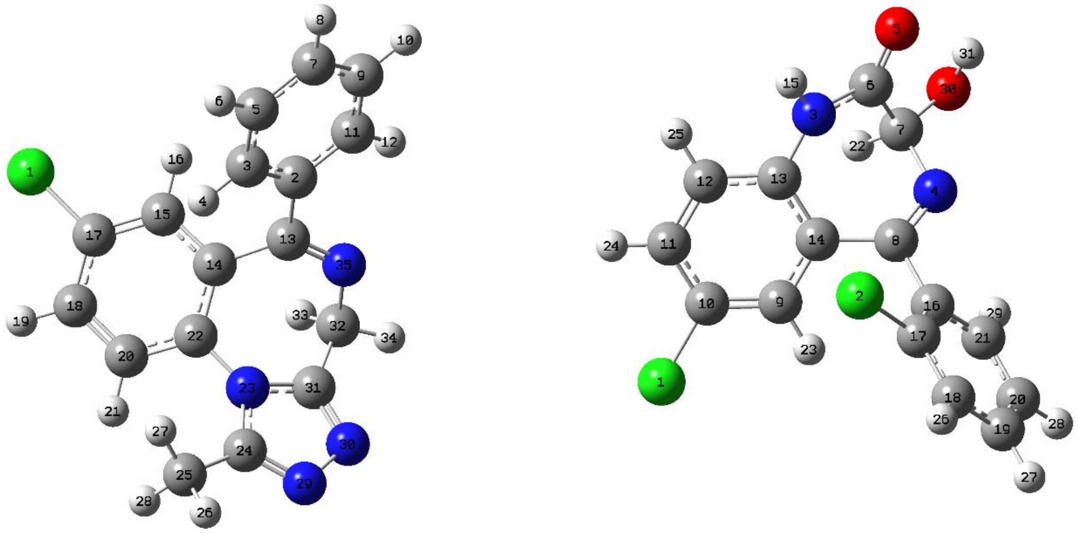

Figure S1. Optimized structures (at M05-2X/6-311++G(d,p) level of theory) of alprazolam (left) and lorazepam (right) with atom enumeration.

Table S1. Experimental and theoretical (optimized using mentioned functionals in conjunction with 6-311++G(d,p) basis set) bond lengths of lorazepam (atom numeration follows Figure S1)

Bond	EXP.	B3LYP	B3LYP-D3BJ	B3PW91	CAM-B3LYP	M05-2X	M06-2X
Cl1-C10	1.728	1.755	1.753	1.742	1.744	1.738	1.740
C10-C9	1.382	1.384	1.383	1.383	1.378	1.380	1.381
C9-C14	1.401	1.403	1.402	1.401	1.397	1.397	1.399

C10-C11	1.361	1.394	1.394	1.393	1.388	1.390	1.392
C11-C12	1.365	1.386	1.385	1.384	1.380	1.382	1.383
C12-C13	1.396	1.401	1.400	1.399	1.394	1.396	1.398
C13-C14	1.378	1.410	1.407	1.407	1.400	1.400	1.402
C14-C8	1.471	1.489	1.485	1.484	1.487	1.485	1.488
C8-C16	1.493	1.498	1.492	1.493	1.494	1.491	1.494
C16-C17	1.371	1.401	1.400	1.399	1.394	1.394	1.396
C17-Cl2	1.737	1.762	1.760	1.747	1.750	1.744	1.747
C17-C18	1.382	1.392	1.391	1.390	1.386	1.387	1.389
C18-C19	1.392	1.392	1.391	1.389	1.386	1.388	1.389
C19-C20	1.393	1.393	1.393	1.391	1.388	1.390	1.391
C20-C21	1.392	1.390	1.389	1.388	1.385	1.386	1.388
C21-C16	1.413	1.402	1.400	1.399	1.395	1.395	1.397
C8-N4	1.272	1.279	1.278	1.278	1.270	1.271	1.272
N4-C7	1.446	1.454	1.454	1.448	1.448	1.451	1.453
C7-C6	1.501	1.537	1.535	1.532	1.529	1.529	1.533
C6-O5	1.240	1.215	1.214	1.213	1.210	1.209	1.207
C6-N3	1.313	1.373	1.371	1.368	1.365	1.366	1.370
C7-O30	1.404	1.408	1.389	1.384	1.383	1.383	1.381
N3-C13	1.406	1.390	1.406	1.403	1.406	1.404	1.405
R		0.99	0.99	0.99	0.99	0.99	0.99
MAE [Å]		0.017	0.015	0.014	0.014	0.013	0.014

Table S2. Experimental and theoretical (optimized using mentioned functionals in conjunction with 6-311++G(d,p) basis set) bond angles of lorazepam (atom numeration follows Figure S1)

Bond	EXP.	B3LYP	B3LYP-D3BJ	B3PW91	CAM-B3LYP	M05-2X	M06-2X
Cl1-C10-C9	118.54	119.71	119.69	119.78	119.75	119.71	119.72
Cl1-C10-C11	120.51	119.53	119.51	119.57	119.57	119.46	119.50
C10-C9-C14	119.80	120.68	120.49	120.65	120.59	120.20	120.30
C9-C14-C8	118.80	119.31	119.07	119.25	119.23	119.07	119.11
C9-C14-C13	119.24	118.72	118.96	118.86	118.90	119.27	119.20
C14-C13-C12	118.93	119.60	119.58	119.53	119.64	119.64	119.59
C14-C13-N3	122.46	122.08	121.92	121.98	121.98	121.74	121.79
C13-C12-C11	121.65	121.10	120.99	121.08	120.97	120.76	120.84
C12-C11-C10	119.21	119.11	119.15	119.20	119.19	119.28	119.25
C14-C8-C16	117.10	118.38	117.58	118.30	118.16	117.46	117.49
C14-C8-N4	125.57	125.11	125.24	125.07	124.96	125.15	125.20
C8-C16-C17	123.57	124.07	123.59	124.01	123.81	123.39	123.41
C16-C17-Cl2	119.50	120.92	120.80	120.92	120.87	120.84	120.81
C16-C17-C18	124.06	121.50	121.45	121.35	121.38	121.27	121.31

Cl2-C17-C18	116.42	117.52	117.70	117.67	117.71	117.83	117.82
C17-C18-C19	117.77	119.63	119.53	119.68	119.64	119.52	119.52
C18-C19-C20	120.94	120.03	120.12	120.07	120.04	120.17	120.16
C19-C20-C21	119.23	119.75	119.75	119.72	119.75	119.75	119.75
C20-C21-C16	121.00	121.41	121.24	121.39	121.27	121.01	121.09
C21-C16-C17	116.98	117.66	117.89	117.78	117.90	118.25	118.14
C21-C16-C8	119.43	118.27	118.52	118.20	118.28	118.35	118.45
C16-C8-N4	117.32	116.42	117.11	116.53	116.78	117.31	117.23
C8-N4-C7	118.04	119.84	119.11	119.44	119.71	118.39	118.85
N4-C7-C6	108.07	107.73	107.34	107.54	107.59	106.80	107.00
C7-C6-O5	119.65	121.99	121.95	121.85	121.61	121.85	121.84
C7-C6-N3	117.99	115.05	115.05	115.07	115.32	115.07	115.10
O5-C6-N3	122.34	122.96	122.99	123.07	123.07	123.07	123.05
C6-N3-C13	125.80	127.38	127.24	127.11	126.95	126.61	126.95
N4-C7-O30	110.19	111.69	111.78	111.83	111.76	111.74	111.74
O30-C7-C6	110.30	110.16	110.15	109.91	109.87	109.81	110.07
R		0.95	0.96	0.95	0.96	0.96	0.96
MAE [°]		1.04	0.94	1.02	0.99	0.91	0.92

Table S3. Experimental and theoretical (optimized using mentioned functionals in conjunction with 6-311++G(d,p) basis set) bond lengths of alprazolam (atom numeration follows Figure S1)

Bond	EXP.	B3LYP	B3LYP-D3BJ	B3PW91	CAM-B3LYP	M05-2X	M06-2X
Cl1-C17	1.737	1.754	1.753	1.741	1.743	1.737	1.739
C17-C18	1.383	1.392	1.392	1.391	1.386	1.388	1.390
C18-C20	1.389	1.388	1.387	1.385	1.382	1.383	1.384
C20-C22	1.394	1.398	1.397	1.396	1.392	1.393	1.395
C22-C14	1.417	1.410	1.407	1.407	1.401	1.401	1.403
C14-C15	1.382	1.403	1.401	1.400	1.396	1.397	1.399
C15-C17	1.378	1.387	1.386	1.385	1.381	1.382	1.384
C22-N23	1.422	1.417	1.414	1.411	1.414	1.410	1.413
N23-C24	1.378	1.388	1.387	1.383	1.380	1.379	1.380
C24-C25	1.479	1.491	1.488	1.486	1.487	1.488	1.489
C24-N29	1.321	1.308	1.308	1.307	1.300	1.301	1.302
N29-N30	1.383	1.383	1.386	1.373	1.375	1.371	1.374
N30-C31	1.297	1.301	1.302	1.300	1.294	1.295	1.296
C31-N23	1.374	1.384	1.385	1.379	1.376	1.375	1.377
C31-C32	1.484	1.490	1.488	1.485	1.486	1.486	1.488
C32-N35	1.470	1.465	1.464	1.458	1.458	1.460	1.461

N35-C13	1.272	1.280	1.279	1.278	1.271	1.271	1.273
C13-C14	1.503	1.504	1.499	1.498	1.500	1.498	1.502
C13-C2	1.481	1.495	1.490	1.489	1.492	1.490	1.494
C2-C3	1.388	1.401	1.400	1.399	1.394	1.395	1.397
C3-C5	1.378	1.393	1.392	1.391	1.388	1.389	1.391
C5-C7	1.382	1.393	1.393	1.391	1.387	1.389	1.390
C7-C9	1.388	1.396	1.395	1.394	1.390	1.391	1.393
C9-C11	1.371	1.390	1.389	1.388	1.385	1.386	1.388
C11-C2	1.392	1.402	1.401	1.400	1.395	1.395	1.397
R		0.99	0.99	0.99	0.99	0.99	0.99
MAE [Å]		0.009	0.009	0.008	0.007	0.007	0.008

Table S4. Experimental and theoretical (optimized using mentioned functionals in conjunction with 6-311++G(d,p) basis set) bond angles of alprazolam (atom numeration follows Figure S1)

	EXP.	B3LYP	B3LYP-D3BJ	B3PW91	CAM-B3LYP	M05-2X	M06-2X
Cl1-C17-C18	119.37	119.57	119.54	119.62	119.60	119.47	119.49
Cl1-C17-C15	119.26	119.55	119.51	119.60	119.59	119.52	119.54
C17-C18-C20	119.34	118.99	119.00	119.06	119.06	119.08	119.06
C18-C20-C22	119.38	120.94	120.78	120.90	120.80	120.53	120.60
C20-C22-C14	121.26	120.12	120.22	120.13	120.22	120.41	120.40
C20-C22-N23	119.31	119.64	119.71	119.72	119.60	119.62	119.52
N23-C22-C14	119.41	120.22	120.05	120.14	120.15	119.94	120.07
C22-C14-C15	117.59	118.25	118.41	118.33	118.34	118.52	118.43
C22-C14-C13	122.73	123.38	123.27	123.32	123.34	123.25	123.25
C14-C15-C17	121.05	120.82	120.62	120.80	120.76	120.43	120.54
C15-C17-C18	121.36	120.87	120.96	120.78	120.81	121.01	120.97
C22-N23-C24	130.64	131.40	130.99	131.43	131.33	131.26	131.02
C22-N23-C31	123.84	124.26	124.42	124.18	124.29	124.29	124.61
N23-C24-C25	126.66	126.03	125.56	125.99	125.93	125.63	125.45
C25-C24-N29	124.95	124.57	125.13	124.70	124.70	125.06	125.14
C24-N29-N30	108.53	108.53	108.48	108.61	108.54	108.59	108.53
N29-N30-C31	107.32	107.40	107.47	107.44	107.42	107.49	107.45
N30-C31-N23	110.38	110.52	110.30	110.43	110.48	110.37	110.44
C30-C31-C32	129.32	128.86	129.31	129.11	128.90	129.35	129.21
C31-C32-N35	108.75	109.64	109.32	109.45	109.51	108.95	109.23
C32-N35-C13	118.03	119.73	119.23	119.27	119.72	118.62	119.08
N35-C13-C14	125.40	124.93	125.11	124.93	124.89	125.13	125.21
N35-C13-C2	117.67	117.39	117.80	117.48	117.60	117.96	117.79
C14-C13-C2	116.90	117.66	117.08	117.58	117.49	116.89	116.98
C13-C2-C3	120.98	121.60	121.34	121.56	121.56	121.31	121.38

C2-C3-C5	121.35	120.58	120.48	120.51	120.50	120.28	120.35
C3-C5-C7	120.01	120.11	120.07	120.09	120.08	120.04	120.05
C5-C7-C9	119.05	119.71	119.78	119.76	119.76	119.89	119.84
C7-C9-C11	120.73	120.25	120.22	120.23	120.20	120.18	120.19
C9-C11-C2	120.76	120.51	120.40	120.45	120.44	120.21	120.28
C11-C2-C3	118.02	118.83	119.04	118.94	119.02	119.40	119.27
C11-C2-C13	120.90	119.51	119.58	119.44	119.38	119.25	119.30
R		0.99	0.99	0.99	0.99	0.99	0.99
MAE [°]		0.59	0.52	0.56	0.57	0.50	0.52

Table S5. Experimental, theoretical (at M05-2X/6-311++G(d,p) level of theory), and predicted (by NMRDB predictor) ^{13}C NMR chemical shifts (in ppm) of lorazepam (atom numeration follows Figure S1)

Carbon atom	Experimental	Theoretical	Predicted
C6	169.4	162.9	170.1
C7	83.1	75.9	82.6
C8	162.3	164.8	165.8
C14	127.1	130.3	127.3
C9	130	128.6	129.4
C10	127.9	133.1	133.1
C11	132.1	132.6	128.9
C12	123.4	122.6	120.11
C13	138	133.6	136.4
C16	137.4	138.5	137.9
C17	132.1	137.4	132
C18	129	129.5	129.2
C19	131.7	132.1	128.6
C20	127.7	127.7	128.6
C21	131.6	132.1	130.5
R		0.98	0.99
MAE [ppm]		2.63	1.64

Table S6. The assigned vibrations of LOR (at M05-2X/6-31+G(d,p) level of theory) (Abbreviations: ν – stretching, β – deformation in plane, γ – deformation out of plane, τ – torsion, ph – phenyl rings, diaz – diazepine ring)

Mode of vibration	Experimental wavenumber [cm^{-1}]	Theoretical wavenumber [cm^{-1}]		Theoretical IR intensity	[a]PED ≥ 5%
	FT-IR	Unscaled	Scaled		
1	3390	3796	3454	100.1	υ_{OH}(99)

2	3335	3661	3331	71.9	υ_{NH}(99)
3		3284	2988	0.2	υ_{CH}(93) ph
4	2975	3283	2988	6.6	υ_{CH}(93) ph
5		3279	2983	2.1	υ_{CH}(93) ph
6		3271	2977	0.3	υ_{CH}(93) ph
7		3270	2975	4.7	υ_{CH}(93) ph
8		3258	2965	1.6	υ_{CH}(93) ph
9	2936	3255	2962	3.8	υ_{CH}(93) ph
10	2846	3096	2817	11.8	υ_{CH}(99) diaz
11	1660	1828	1664	1639.1	υ_{CO}(79) + δ_{NCC} diaz
12		1751	1594	224.4	$\upsilon_{C=N}$(72) diaz
13		1697	1544	2.6	υ_{CC}(64) ph
14		1692	1539	31.1	υ_{CC}(80) ph
15		1666	1516	42.4	υ_{CC}(54) ph
16		1663	1514	9.4	υ_{CC}(70) ph
17	1463	1553	1413	330.1	δ_{CCH}(26) ph + υ_{NC}(8) diaz
18	1426	1539	1400	87.1	δ_{CCH}(26) ph + υ_{CC}(19) ph
19	1382	1515	1379	92.7	δ_{CNH}(23) + υ_{NC}(19) + υ_{CC}(5) diaz
20	1369	1497	1362	87.9	δ_{CCH}(48) ph
21	1340	1474	1342	109.7	δ_{CHO}(59) diaz + δ_{CCH}(10) ph
22	1321	1434	1305	4.4	δ_{CNH}(18) diaz + δ_{CCH}(15) ph + υ_{CC}(14) ph
23	1296	1389	1264	261.4	δ_{NCH}(39) + δ_{HCl}(18) + υ_{NC}(14) diaz
24	1259	1380	1255	99.0	υ_{CC}(34) diaz + δ_{COH}(15) diaz
25		1351	1230	90.9	δ_{COH}(30) diaz + υ_{CC}(14) ph + υ_{CN}(7) diaz
26	1222	1344	1223	29.5	υ_{CN}(9) diaz + υ_{CC}(13) ph + δ_{COH}(8) diaz
27	1204	1338	1218	293.7	υ_{CC}(51) ph
28		1297	1180	28.2	δ_{CCH}(28) ph + υ_{CC}(16) ph
29		1293	1176	69.9	δ_{CCH}(42) ph
30	1169	1273	1158	156.2	δ_{CCH}(27) diaz + υ_{CO}(11) diaz + υ_{CN}(9) diaz + δ_{CHN}(10) diaz
31	1144	1256	1143	72.5	υ_{CN}(13) diaz + υ_{CC}(17) ph + δ_{CNH}(5) diaz
32	1116	1217	1108	90.3	υ_{CC}(27) ph + δ_{CCH}(5) ph
33	1096	1197	1090	866.5	υ_{CO}(32) diaz + δ_{COH}(9) diaz + δ_{CCH}(14) ph
34	1080	1192	1085	80.3	δ_{CCH}(30) ph + υ_{CO}(13) diaz
35	1071	1172	1066	26.9	δ_{CCH}(24) phen + υ_{CC}(6) ph
36	1059	1168	1063	31.9	δ_{CCH}(32) phen + υ_{CC}(12) ph
37	1036	1143	1040	127.5	υ_{CC}(15) ph + υ_{CCl}(14) diaz + δ_{CCH}(20) ph
38	1002	1091	992	20.3	δ_{CCH}(25) ph + δ_{CCC}(11) ph + υ_{CCl}(10) diaz
39		1076	979	94.2	υ_{CN}(18) diaz + υ_{CC}(24) ph
40	984	1071	975	147.6	υ_{CN}(26) diaz + υ_{CC}(21) ph + δ_{CNC}(5) diaz
41		1029	937	0.6	γ_{HCCC}(100) ph
42	915	996	906	6.8	γ_{HCCC}(100) ph
43	900	994	904	0.5	γ_{HCCC}(100) ph
44	890	969	882	37.5	υ_{CN}(13) diaz + δ_{CCC}(6) ph + δ_{NCC}(5) diaz
45	879	945	860	90.5	γ_{HCCC}(14) ph + υ_{CC}(11) ph + δ_{NCC}(5) diaz + δ_{NCO}(5) diaz

46		930	846	49.6	γ_{HCCC}(81) ph
47		907	825	1.4	γ_{HCCC}(100) ph
48		883	804	18.6	υ_{CC}(12) ph + γ_{OCNC} (14) diaz + δ_{NCC}(8) diaz + τ_{OCCO}(5) diaz
49	779	857	780	189.2	γ_{HCCC}(62) ph + τ_{CCCN}(5) diaz
50		839	763	128.5	γ_{HCCC}(20) ph + υ_{CCl}(7) ph + γ_{OCNC}(6) diaz
51	735	798	727	98.3	γ_{HCCC}(52) ph + γ_{CCNC}(11) diaz
52	717	779	709	159.0	τ_{CCCN}(17) diaz + γ_{CCNC}(17) diaz + γ_{HCCC}(11) ph + τ_{CCNC}(9) diaz
53		754	686	114.1	υ_{CCl}(11) ph + δ_{CCC}(8) ph + γ_{ClCCC}(5) ph + γ_{CCCC}(11) ph
54		746	678	42.5	γ_{CCCC}(17) ph + γ_{ClCCC}(15) ph + τ_{CCNC}(8) diaz + τ_{CCCC}(8) diaz
55	673	733	667	91.4	δ_{CHN}(16) diaz + γ_{OCNC}(7) diaz + δ_{CCO}(5) diaz
56	631	717	652	92.1	υ_{CCl}(12) ph + δ_{CCC}(6) diaz + δ_{CNC}(6) diaz
57		692	629	66.2	δ_{CCC}(18) ph + δ_{CCC}(6) ph + γ_{OCNC}(6) ph
58		675	614	17.0	τ_{CCCC}(23) ph + γ_{CCNC}(10) diaz + γ_{ClCCC}(9) ph
59	601	653	595	17.5	δ_{CCC}(39) ph
60	565	618	562	40.2	δ_{CNC}(12) diaz + δ_{NCO}(7) diaz + δ_{CCC}(12) ph
61	548	555	505	448.0	δ_{CNH}(26) diaz + δ_{CNC}(10) diaz + δ_{NCO}(12) diaz + υ_{CCl}(7) ph
62		550	501	355.6	γ_{ClCCC}(39) ph + δ_{CNH}(16) diaz + γ_{CCCN}(7) diaz
63		533	485	12.5	τ_{CCCC}(15) ph + δ_{CNH}(20) diaz + γ_{ClCCC}(7) ph
64		522	475	28.2	δ_{CNH}(29) diaz + τ_{CCCC}(10) ph
65		484	441	35.1	γ_{ClCCC}(10) ph + τ_{ClCCC}(10) ph + τ_{CCCN}(8) ph + τ_{CCCC}(8) ph
66		470	428	37.4	τ_{CCCC}(23) ph + γ_{ClCCC}(13) ph + υ_{CCl}(12) ph
67		450	410	26.0	υ_{CCl}(13) ph + τ_{CCCC}(22) ph + γ_{ClCCC}(6) ph
68		427	388	517.3	τ_{HOCN} (51) ph + δ_{CCO}(15) diaz+ υ_{CCl}(4) ph
69		418	380	103.5	τ_{CCCC}(9) ph + τ_{ClCCC}(8) ph + υ_{CC}(7) ph + δ_{NCC}(6) diaz
70		396	360	163.4	δ_{ClCC}(17) ph + δ_{ClCC}(10) ph + δ_{NCO}(7) diaz
71		388	353	119.1	γ_{OCNC}(12) diaz + δ_{CNC} (7) diaz + γ_{HOCN}(6) diaz + δ_{ClCC}(6) ph
72		383	349	26.7	τ_{CCCC}(10) ph + τ_{CCNC}(10) diaz + τ_{CNCC}(6) diaz + δ_{NCC}(9) diaz
73		342	311	199.0	τ_{HOCN}(11) diaz + δ_{ClCC}(9) ph + τ_{CCCCl}(11) ph + τ_{NCCC}(7) ph
74		330	300	119.9	υ_{CCl}(10) ph + τ_{NCCC}(7) diaz + δ_{ClCC}(6) ph + υ_{CN}(6) diaz
75		306	278	172.0	τ_{HOCN}(11) diaz + δ_{NCC}(9) diaz + δ_{CCO} (8) diaz + δ_{ClCC}(7) ph

76		281	255	78.2	τ_{CCCC}(10) ph + τ_{CCCCl}(8) diaz + δ_{CCC} (7) ph + τ_{OCNC}(7) diaz
77		225	205	83.1	τ_{OCNC}(37) diaz + τ_{OCCO}(9) diaz + τ_{CCNC}(7) diaz + δ_{ClCC}(7) ph
78		222	202	8.9	υ_{CC}(16) ph + δ_{CCC}(8) ph + δ_{ClCC}(6) ph + δ_{NCC}(6) diaz
79		207	188	35.4	τ_{CCCCl}(15) ph + δ_{ClCC}(19) ph + τ_{ClCCC}(7) diaz
80		188	171	7.9	τ_{CCCC}(13) ph + δ_{CCC}(21) ph + δ_{ClCC}(11) ph
81		147	133	8.2	τ_{CCNC}(21) diaz + τ_{CCCCl}(37) ph + τ_{CCCC}(7) ph
82		132	120	15.5	τ_{CCNC}(21) diaz + τ_{CCCCl}(21) ph + τ_{OCNC}(7) diaz
83		101	92	11.9	τ_{OCCO}(24) diaz + τ_{OCNC}(18) diaz + τ_{CCNC}(7) diaz + τ_{CCCN}(7) diaz
84		66	60	69.9	τ_{OCNC}(16) diaz + τ_{CCCN}(11) diaz + τ_{CCCC}(9) diaz + τ_{NCCC}(9) diaz
85		60	55	63.1	γ_{CCNC}(30) diaz + τ_{CCCC}(14) ph + δ_{CCC}(10) ph + τ_{CCCN}(8) diaz
86		46	41	94.6	τ_{CCCN}(26) diaz + τ_{CCCC}(8) ph + τ_{ClCCC}(8) ph + δ_{CCC}(7) ph
87		28	25	88.2	τ_{CCCN}(79) diaz
MAE [cm^{-1}]			13		

Table S7. The calculated Bond Critical Points (BCP) properties at the M05-2X/6-31+G(d,p) level of theory: the electron density (ρ(r)) and its Laplacian ($\nabla^2\rho(r)$); the Lagrangian kinetic electron density (G(r)) and the potential electron density (V(r)); the density of the total energy of electrons (H(r)) – Cremer-Kraka electronic energy density; the interatomic bond energy, E_{bond}, for the interactions between LOR and adulterants/diluents (these interactions are depicted in the main text)

Bond	ρ(r) [a.u.]	$\nabla^2\rho(r)$ [a.u.]	G(r) [kJ mol^{-1}]	V(r) [kJ mol^{-1}]	H(r) [kJ mol^{-1}]	-G(r)/V(r)	E_{bond} [kJ mol^{-1}]
**LOR dimer 1**							
O−H···O_{carb} (**1**)	0.024	0.088	53.8	-50.2	3.6	1.1	-25.1
O_{carb}···O_{carb} (**2**)	0.006	0.021	12.3	-10.9	1.4	1.1	-5.4
O_{carb}···H−O (**3**)	0.024	0.088	53.7	-50.1	3.6	1.1	-25.1
N···N (**4**)	0.001	0.004	2.1	-1.2	0.9	1.7	-0.6
Cl···C_{phen} (**5**)	0.005	0.014	7.8	-6.0	1.7	1.3	-3.0
C_{phen}···C_{phen} (**6**)	0.004	0.012	6.7	-5.4	1.4	1.3	-2.7
C_{phen}···Cl (**7**)	0.005	0.014	7.8	-6.0	1.7	1.3	-3.0
**LOR dimer 2**							
$O_{hydroxyl}$···H (**1**)	0.005	0.021	11.3	-9.2	2.2	1.2	-4.6
H···Cl (**2**)	0.010	0.034	18.0	-13.7	4.2	1.3	-6.9
C_{phen}···Cl (**3**)	0.007	0.023	12.5	-9.3	3.2	1.3	-4.7
C_{diaz}···Cl (**4**)	0.006	0.022	11.6	-8.9	2.7	1.3	-4.4
Cl···H−N (**5**)	0.008	0.028	14.9	-11.5	3.3	1.3	-5.8
Cl···H−C_{phen} (**6**)	0.003	0.010	5.1	-3.5	1.6	1.5	-1.8

H···C_{phen} (**7**)	0.002	0.007	3.3	-2.3	1.1	1.5	-1.1
H···Cl (**8**)	0.006	0.020	10.0	-6.9	3.1	1.5	-3.4

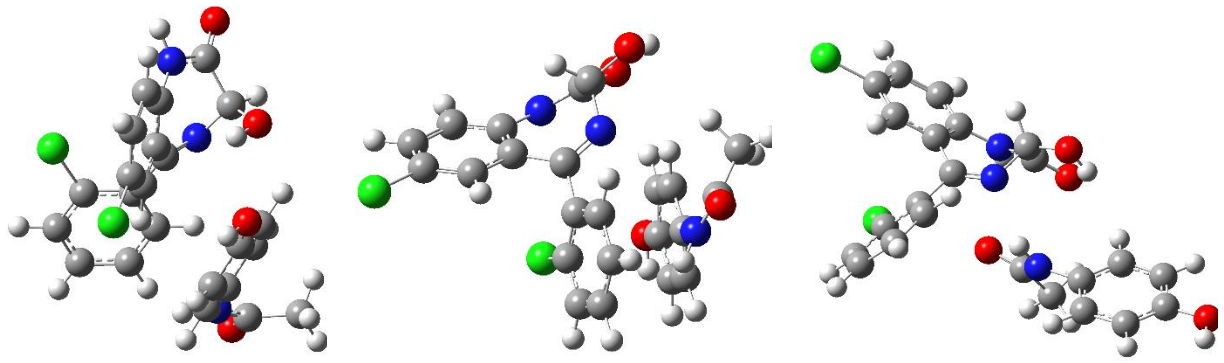

(**a**) (**b**) (**c**)

Figure S2. (**a**–**c**) The most stable structures (at M05-2X/6-31+G(d,p) level of theory) formed between LOR and paracetamol.

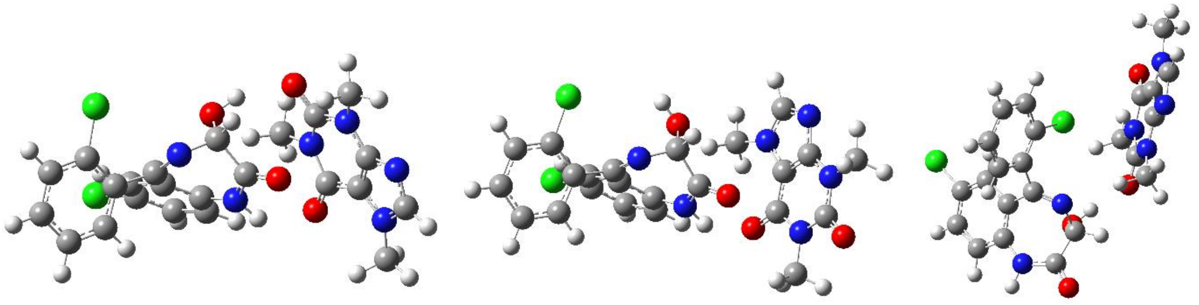

(**a**) (**b**) (**c**)

Figure S3. (**a**–**c**) The most stable structures (at M05-2X/6-31+G(d,p) level of theory) formed between LOR and caffeine.

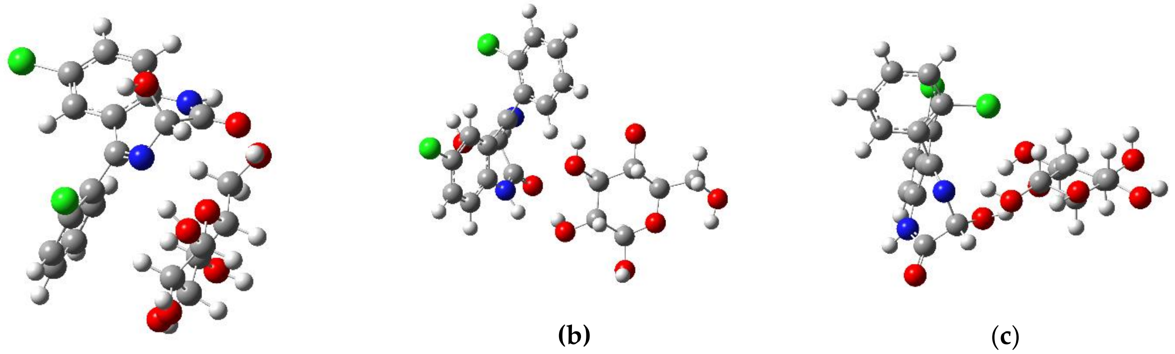

(**b**) (**c**)

(**a**)

Figure S4. (**a**–**c**) The most stable structures (at M05-2X/6-31+G(d,p) level of theory) formed between LOR and glucose.

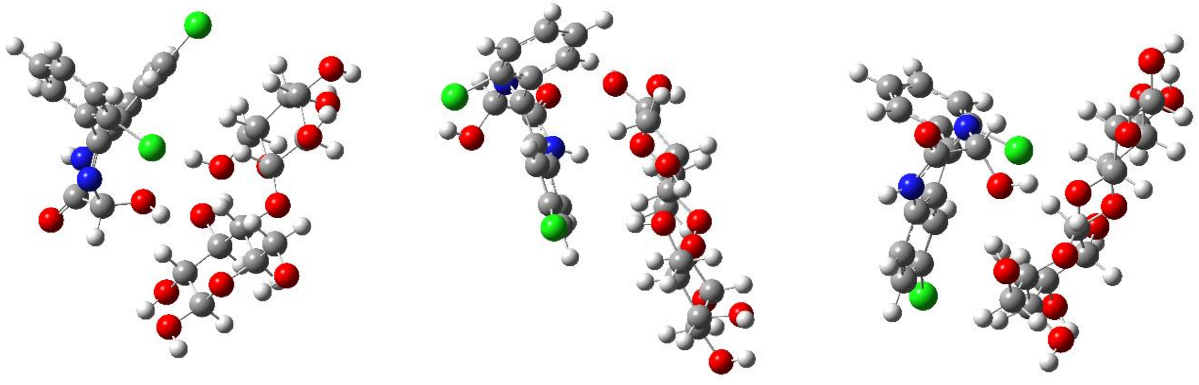

(**a**) (**b**) (**c**)

Figure S5. (**a**–**c**) The most stable structures (at M05-2X/6-31+G(d,p) level of theory) formed between LOR and lactose.

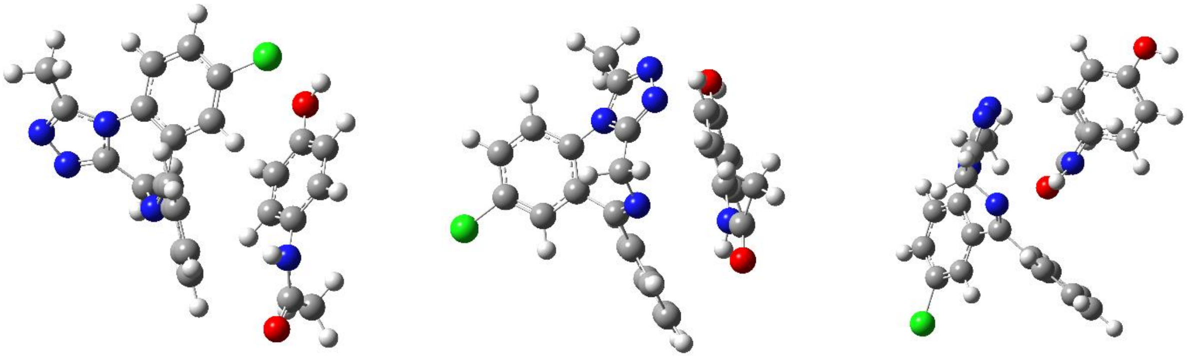

(**a**) (**b**) (**c**)

Figure S6. (**a**–**c**) The most stable structures (at M05-2X/6-31+G(d,p) level of theory) formed between ALP and paracetamol.

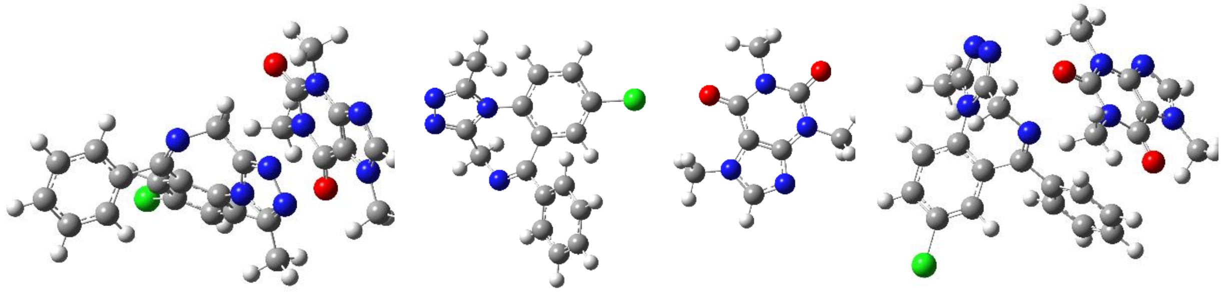

(**a**) (**b**) (**c**)

Figure S7. (**a**–**c**) The most stable structures (at M05-2X/6-31+G(d,p) level of theory) formed between ALP and caffeine.

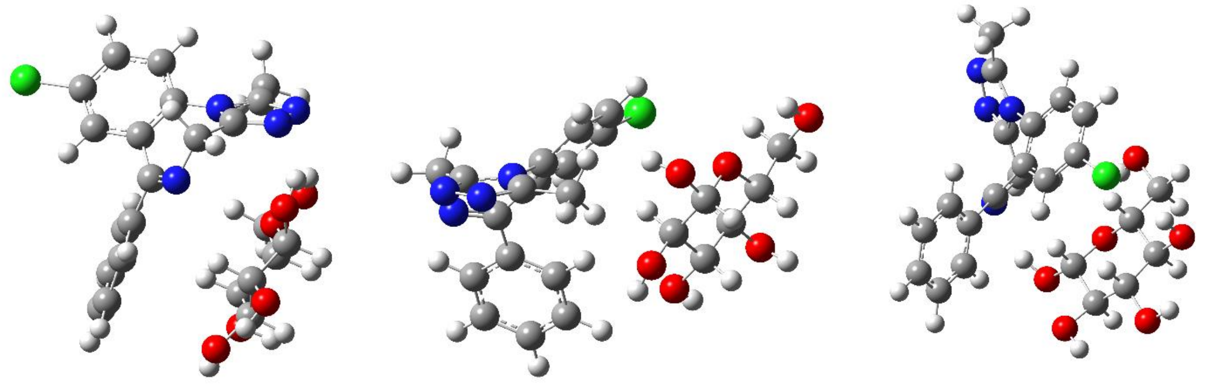

(**a**) **(b)** (**c**)

Figure S8. (**a**–**c**) The most stable structures (at M05-2X/6-31+G(d,p) level of theory) formed between ALP and glucose.

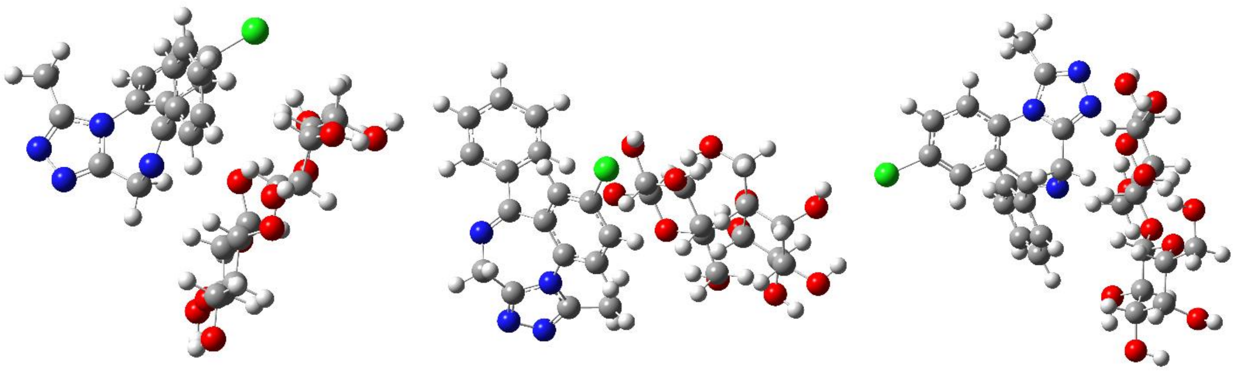

(**a**) **(b)** (**c**)

Figure S9. (**a**–**c**) The most stable structures (at M05-2X/6-31+G(d,p) level of theory) formed between LOR and lactose.

Table S8. The calculated Bond Critical Points (BCP) properties at the M05-2X/6-31+G(d,p) level of theory: the electron density (ρ(r)) and its Laplacian (∇^2ρ(r)); the Lagrangian kinetic electron density (G(r)) and the potential electron density (V(r)); the density of the total energy of electrons (H(r)) – Cremer-Kraka electronic energy density; the interatomic bond energy, E_{bond}, for the interactions between LOR and adulterants/diluents (these interactions are depicted in the main text)

Bond	ρ(r) [a.u.]	∇^2ρ(r) [a.u.]	G(r) [kJ mol^{-1}]	V(r) [kJ mol^{-1}]	H(r) [kJ mol^{-1}]	-G(r)/V(r)	E_{bond} [kJ mol^{-1}]
**LOR···paracetamol**							
O_{carb}···H−C (**1**)	0.009	0.033	19.4	-17.2	2.2	1.1	-8.6
O_{carb}···C (**2**)	0.008	0.031	17.4	-14.7	1.2	1.2	-7.3
N···H−N (**3**)	0.019	0.056	36.0	-35.4	1.0	1.0	-17.7
N···O_{carb} (**4**)	0.008	0.023	14.4	-13.4	1.1	1.1	-6.7
N_{amino}···O_{carb} (**5**)	0.009	0.028	17.2	-16.0	1.1	1.1	-8.0
Cl···O_{carb} (**6**)	0.005	0.021	11.3	-8.9	1.3	1.3	-4.5
**LOR···caffeine**							
C−H···O_{carb} (**1**)	0.006	0.022	12.4	-10.0	2.3	1.2	-5.0
N−H···O (**2**)	0.010	0.034	20.3	-17.9	2.3	1.1	-9.0
N···H−C (**3**)	0.007	0.025	14.4	-12.3	2.1	1.2	-6.1
O_{carb}···C (**4**)	0.008	0.026	15.1	-13.3	1.8	1.1	-6.6
O_{carb}···C (**5**)	0.010	0.035	20.6	-18.2	2.5	1.1	-9.1
$O_{hydroxyl}$···H−C (**6**)	0.011	0.034	20.9	-19.3	1.6	1.1	-9.7
$O_{hydroxyl}$−H···O (**7**)	0.024	0.076	50.5	-51.2	-0.7	1.0	-25.6
**LOR···glucose**							
O_{carb}···H−O (**1**)	0.022	0.066	45.0	-46.4	-1.4	1.0	-23.2
O_{carb}···O (**2**)	0.007	0.025	14.6	-12.9	1.8	1.1	-6.4
N−H···O (**3**)	0.012	0.043	25.8	-23.4	2.4	1.1	-11.7
N···O (**4**)	0.008	0.026	16.0	-15.0	1.0	1.1	-7.5
N···H−C (**5**)	0.032	0.085	56.5	-57.2	-0.7	1.0	-28.6
C_{phen}···H−C (**6**)	0.007	0.023	12.6	-10.1	2.5	1.2	-5.1
C_{phen}···H−C (**7**)	0.005	0.016	8.2	-6.0	2.1	1.4	-3.0
C_{phen}···O (**8**)	0.003	0.010	5.3	-3.9	1.4	1.4	-1.9
**LOR···lactose**							
C_{diaz}−H···H (**1**)	0.017	0.060	2.7	-1.7	1.0	1.6	-0.8
$O_{hydroxyl}$−H ···O (**2**)	0.044	0.135	90.8	-93.0	-2.2	1.0	-46.5
$O_{hydroxyl}$···H (**3**)	0.006	0.023	12.5	-10.2	2.3	1.2	-5.1
$O_{hydroxyl}$···H (**4**)	0.014	0.053	30.6	-26.3	4.3	1.2	-13.2
$O_{hydroxyl}$···H (**5**)	0.010	0.034	20.0	-17.7	2.3	1.1	-8.8
$O_{hydroxyl}$···O (**6**)	0.007	0.026	16.2	-15.1	1.1	1.1	-7.5
Cl···H (**7**)	0.004	0.013	6.2	-4.1	2.1	1.5	-2.1
Cl···H (**8**)	0.007	0.025	13.0	-9.4	3.5	1.4	-4.7
Cl···H (**9**)	0.003	0.009	3.1	-3.1	0.0	1.0	-1.6
Cl···O (**10**)	0.005	0.021	11.2	-8.5	2.7	1.3	-4.2
C_{phen}···H (**11**)	0.007	0.011	12.1	-9.7	2.4	1.2	-4.9
C_{phen}···H (**12**)	0.004	0.012	6.4	-4.7	1.6	1.3	-2.4
C_{phen}···H (**13**)	0.004	0.22	3.2	-4.3	-1.0	0.8	-2.1
Cl···H (**14**)	0.003	0.011	5.8	-4.2	1.6	1.4	-2.1
Cl···H (**15**)	0.005	0.018	9.4	-6.7	2.7	1.4	-3.3

Table S9. The calculated Bond Critical Points (BCP) properties at the M05-2X/6-31+G(d,p) level of theory: the electron density (ρ(r)) and its Laplacian ($\nabla^2\rho(r)$); the Lagrangian kinetic electron density (G(r)) and the potential electron density (V(r)); the density of the total energy of electrons (H(r)) – Cremer-Kraka electronic energy density; the interatomic bond energy, E_{bond}, for the interactions between ALP and adulterants/diluents (these interactions are depicted in the main text)

Bond	ρ(r) [a.u.]	$\nabla^2\rho(r)$ [a.u.]	G(r) [kJ mol^{-1}]	V(r) [kJ mol^{-1}]	H(r) [kJ mol^{-1}]	-G(r)/V(r)	E_{bond} [kJ mol^{-1}]
**ALP···paracetamol**							
N···H (**1**)	0.014	0.041	25.8	-24.5	1.3	1.1	-12.3
C_{phen}–H···O (**2**)	0.008	0.032	17.5	-14.1	3.4	1.2	-7.0
C_{diaz}···O_{carb} (**3**)	0.010	0.031	18.6	-16.7	1.9	1.1	-8.4
$N_{triazine}$···O_{carb} (**4**)	0.013	0.040	25.5	-24.6	0.9	1.0	-12.3
$N_{triazine}$···N (**5**)	0.005	0.014	8.5	-7.9	0.6	1.1	-4.0
$N_{triazine}$···H (**6**)	0.011	0032	19.3	-17.4	1.9	1.1	-8.7
$N_{triazine}$···H (**7**)	0.006	0.017	10.2	-8.1	2.1	1.3	-4.1
**ALP···caffeine**							
C_{phen}–H···O_{carb} (**1**)	0.011	0.036	22.0	-20.0	1.9	1.1	-10.0
C_{phen}··· H–C (**2**)	0.006	0.019	10.1	-7.6	2.5	1.3	-3.8
C_{methyl}–H···O_{carb} (**3**)	0.006	0.023	12.9	-10.4	2.5	1.2	-5.2
$C_{triazine}$ ···O_{carb} (**4**)	0.006	0.022	12.8	-10.8	2.0	1.2	-5.4
N_{diaz} ···H–C (**5**)	0.009	0.029	16.6	-14.2	2.4	1.2	-7.1
N_{diaz} ···C (**6**)	0.009	0.025	15.1	-13.6	1.5	1.1	-6.8
C_{diaz} ···N (**7**)	0.006	0.017	10.1	-8.9	1.2	1.1	-4.5
C_{diaz}–H···O_{carb} (**8**)	0.006	0.022	12.1	-9.4	2.7	1.3	-4.7
**ALP···glucose**							
C_{phen}–H···O (**1**)	0.004	0.012	6.7	-5.3	1.4	1.3	-2.6
C_{phen}···H (**2**)	0.004	0.013	6.6	-4.6	2.0	1.4	-2.3
N···H (**3**)	0.007	0.021	11.7	-9.7	1.9	1.2	-4.9
C_{phen}···H (**4**)	0.008	0.024	13.0	-10.5	2.5	1.2	-5.2
C_{phen}···H (**5**)	0.003	0.009	4.6	-3.4	1.2	1.4	-1.7
N···O (**6**)	0.005	0.018	10.7	-9.3	1.3	1.1	-4.7
C_{diaz}–H···O (**7**)	0.007	0.026	14.1	-11.2	2.9	1.3	-5.6
$N_{triazine}$···O (**8**)	0.010	0.031	19.6	-18.8	0.8	1.0	-9.4
$N_{triazine}$···H–O (**9**)	0.024	0.070	46.1	-46.1	-0.1	1.0	-23.1
$N_{triazine}$···H–O (**10**)	0.012	0.038	23.6	-21.9	1.6	1.1	-11.0
**ALP···lactose**							
C_{phen}–H···O (**1**)	0.006	0.024	13.2	-10.5	2.7	1.3	-5.2
C_{phen}···H (**2**)	0.007	0.020	10.6	-8.2	2.4	1.3	-4.1
C_{phen}–H···O (**3**)	0.006	0.021	11.5	-8.8	2.6	1.3	-4.4
C_{phen}–H···H (**4**)	0.004	0.014	6.9	-4.5	2.4	1.5	-2.3
C_{phen}···H (**5**)	0.006	0.019	9.9	-7.4	2.5	1.3	-3.7
N_{diaz}···H (**6**)	0.005	0.015	8.4	-6.6	1.7	1.3	-3.3
N_{diaz} ···H (**7**)	0.010	0.030	17.6	-15.9	1.8	1.1	-7.9
N_{diaz}···O (**8**)	0.00	0.022	13.3	-11.8	1.5	1.1	-5.9
$C_{triazine}$–H···C (**9**)	0.009	0.035	20.1	-17.4	2.7	1.2	-8.7
$N_{triazine}$···O (**10**)	0.005	0.018	10.5	-12.0	-1.5	0.9	-6.0
$N_{triazine}$···H–C (**11**)	0.009	0.029	16.1	-13.4	2.7	1.2	-6.7
$N_{triazine}$···H–O (**12**)	0.016	0.050	32.0	-30.8	1.2	1.0	-15.4
